# Imputation-powered whole-exome analysis identifies genes associated with kidney function and disease in the UK Biobank

**DOI:** 10.1038/s41467-023-36864-8

**Published:** 2023-03-09

**Authors:** Matthias Wuttke, Eva König, Maria-Alexandra Katsara, Holger Kirsten, Saeed Khomeijani Farahani, Alexander Teumer, Yong Li, Martin Lang, Burulca Göcmen, Cristian Pattaro, Dorothee Günzel, Anna Köttgen, Christian Fuchsberger

**Affiliations:** 1 Institute of Genetic Epidemiology, Faculty of Medicine and Medical Center, University of Freiburg, Freiburg, Germany; 2 Renal Division, Department of Medicine, Faculty of Medicine and Medical Center, University of Freiburg, Freiburg, Germany; 3 Eurac Research, Institute for Biomedicine (affiliated to the University of Lübeck), Bolzano, Italy; 4 Institute for Medical Informatics, Statistics and Epidemiology, University of Leipzig, Leipzig, Germany; 5 LIFE Research Centre for Civilization Diseases, University of Leipzig, Leipzig, Germany; 6 Clinical Physiology/Nutritional Medicine, Charité - Universitätsmedizin Berlin, Berlin, Germany; 7 Institute for Community Medicine, University Medicine Greifswald, Greifswald, Germany; 8 DZHK (German Center for Cardiovascular Research), Partner Site Greifswald, Greifswald, Germany; 9 Department of Epidemiology, Johns Hopkins Bloomberg School of Public Health, Baltimore, MD USA

**Keywords:** Chronic kidney disease, Genome-wide association studies

## Abstract

Genome-wide association studies have discovered hundreds of associations between common genotypes and kidney function but cannot comprehensively investigate rare coding variants. Here, we apply a genotype imputation approach to whole exome sequencing data from the UK Biobank to increase sample size from 166,891 to 408,511. We detect 158 rare variants and 105 genes significantly associated with one or more of five kidney function traits, including genes not previously linked to kidney disease in humans. The imputation-powered findings derive support from clinical record-based kidney disease information, such as for a previously unreported splice allele in *PKD2*, and from functional studies of a previously unreported frameshift allele in *CLDN10*. This cost-efficient approach boosts statistical power to detect and characterize both known and novel disease susceptibility variants and genes, can be generalized to larger future studies, and generates a comprehensive resource (https://ckdgen-ukbb.gm.eurac.edu/) to direct experimental and clinical studies of kidney disease.

## Abstract (web summary)

An exome wide association study of UK Biobank data revealed 158 variants and 105 genes significantly associated with kidney function traits and disease. The findings are supported by functional evidence for a previously unreported mutation in CLDN10.

## Introduction

Chronic kidney disease (CKD) is a major public health concern affecting ~10% of the global adult population^1^. Previous genetic studies have either focused on identifying rare, pathogenic variants among patients with suspected monogenic forms of kidney disease, or on using genome-wide association studies (GWAS) in large population-based studies to identify common susceptibility variants for CKD or the most commonly used markers of kidney function and damage, namely, the estimated glomerular filtration rate (eGFR)^2–6^ and the urinary albumin-to-creatinine ratio (UACR)^7–9^. Initial studies of the coding exome or the whole genome to detect rare pathogenic variants and risk genes for kidney dysfunction had limited power^10,11^. These limitations can now be addressed through the availability of whole-exome sequencing (WES) data from the UK Biobank^12^, and the possibility of imputing the samples not sequenced but array genotyped at the time this study started.

As the direct measurement of kidney function is not feasible in population-based studies and clinical routine, the glomerular filtration rate (GFR) is typically estimated from biomarker measurements and demographic information. The most commonly used biomarker for GFR estimation is serum creatinine, a metabolite predominantly produced in skeletal muscle. More recently developed GFR estimating equations also include cystatin C, a protein produced ubiquitously and hence less dependent on muscle mass. Serum urea and urate levels, metabolites mainly produced in the liver, are also strongly reflective of kidney function. Conversely, the UACR reflects damage to the glomerular filter rather than filtration function, by quantifying the leakage of albumin into the urine. Although creatinine and cystatin C have been studied as two of thousands of outcomes in several phenome-wide screens of the coding exome in the UK Biobank^13–17^, a dedicated effort to assess associations with kidney function and disease by leveraging different biomarkers and ICD-based CKD classifications has not been performed. Moreover, the contribution of rare exonic variants independently from common kidney function variants has received little attention. Lastly, previous efforts have stopped short of experimental validation of causal genes or variants.

Here, we use WES-powered imputation in the large white British ancestry sample of the UK Biobank to increase sample size for studies of the coding genome from 166,891 to 408,511. We discover rare variants and genes associated with measures of kidney function and disease, namely creatinine- and cystatin C-based eGFR (“eGFRcrea” and “eGFRcys”, respectively), serum urea (“urea”), serum urate (“urate”), and the UACR in the general population. We validate our findings using the full set of exome sequences from the UK Biobank that was released over the course of this study, characterize our findings across different kidney phenotypes and clinical definitions of CKD, identify cell types and tissues in which the genes are highly expressed, explore the relationship of identified genes across the phenome, and experimentally validate a previously unreported splice allele in *CLDN10* as proof-of-principle for imputation-powered discovery. This comprehensive approach enabled the replication of many known kidney disease-causing variants and genes that serve as positive controls, and the discovery of variants and genes not previously connected to kidney function and disease that represent promising candidates for experimental follow-up. The results are made publicly available as a resource to the scientific community.

## Results

### WES-powered Imputation

Using available WES data of 166,891 samples as a backbone, we imputed genotypes at 7,575,566 variants for an additional 241,620 individuals without WES data, resulting in a total sample size of 408,511 individuals for exome-wide association studies (ExWAS; Methods).

**Fig. 1:**
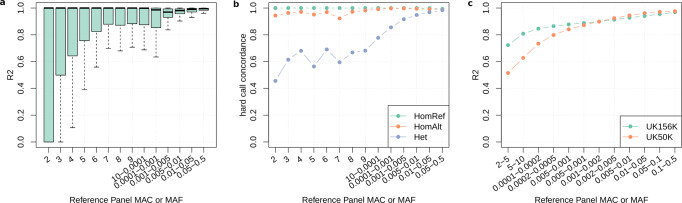
Imputation quality in 10,000 validation samples and 2,191,400 variants. **a** Boxplots of the squared correlation (R2) of sequenced genotypes with imputed dosages. Variants were binned based on their MAC (2–10) or MAF (≥0.0001) in the 156 K reference panel, resulting in a mean of 157 K (27–630 K) variants per bin. The boxes represent the first to the third quartile, the horizontal line the median, and the whiskers extend to 1.5 times the interquartile range. **b** Mean genotype concordance (number of matching genotypes/total number of genotypes) of the hard call imputed genotypes with sequenced data for homozygous reference (HomRef), homozygous alternative (HomAlt), and heterozygous (Het) calls. Variants were binned based on their MAC in the 156 K reference panel, resulting in a mean of 1.5 × 10^9^ (3 × 10^8^–6 × 10^9^) hard calls per bin. **c** Mean squared correlation of sequenced genotypes and imputed dosages using a reference panel of 156 K individuals (this study) and a reference panel of 50 K individuals (Barton et al.). Variants were binned based on the MAC or MAF in their respective reference panel.

Initial imputation performance was evaluated using 10,000 samples with WES data set aside from the imputation reference panel and imputing their genotypes, allowing determination of imputation accuracy for 2,191,400 imputed variants with a minor allele count (MAC) of ≥1. The imputation accuracy at these variants measured as the average squared correlation (R2) between sequenced hard call genotypes and imputed allele dosages showed an overall median of 0.9971 (Q1 = 0.8787, Q3 = 1.0000; mean = 0.8375, sd = 0.3119, Fig. 1a). Very rare variants with MAC 2–5 achieved a median R2 of 0.9999 (*n* = 499,179; Q1 = 0.4995, Q3 = 1.0000; mean = 0.7362, sd = 0.4145).

The concordance (number of matching genotypes/total number of genotypes) of homozygous reference, heterozygous, and homozygous alternative genotypes based on sequenced and imputed data in the validation sample was 0.9998, 0.9778, and 0.9877, respectively (Fig. 1b). Very rare variants with a MAC of 2–5 in the reference panel achieved a concordance of 0.99996, 0.5573, and 0.9569 for homozygous reference, heterozygous, and homozygous alternative calls, respectively. In this low MAC range, the vast majority (>99.9%) of genotypes are (correctly classified) homozygous reference, and true heterozygotes are more frequently imputed as homozygous reference, leading to the relatively low concordance in this genotype class. The true positive rate (TPR) and true negative rate (TNR), computed by defining heterozygous and homozygous alternative genotypes (i.e., carriers of a given variant) as “positive” and homozygous reference genotype (i.e., non-carriers) as “negative”, were 0.9817 and 0.9998, respectively. The false negative rate (carriers incorrectly imputed as non-carriers) was 0.0101, and the false positive rate (non-carriers incorrectly imputed as carriers) was 0.0002.

Together, these comparisons show that study specific WES-imputation enables the estimation of unobserved genotypes with high quality even for very rare variants, providing a solid foundation for subsequent association studies. The imputation quality of our study based on an imputation reference panel of ~167 K individuals was considerably higher than the imputation quality achieved in a previous imputation effort that used a reference panel of ~50 K individuals (Fig. 1c)^16^.

### ExWAS of kidney function traits identifies 158 rare variants

Results from ExWAS of the five continuous kidney markers and 7.5 million variants from 408,511 individuals of European ancestry showed no signs of unaccounted stratification (inflation factor *λ*ː 1.00–1.04, Supplementary Fig. 1; Methods). We identified 174 associations at rare variants of minor allele frequency (MAF) < 1% and MAC ≥ 5 that were significantly associated with at least one kidney phenotype (*p* < 6.8 × 10^−9^; Supplementary Fig. 2, Methods). These associations corresponded to 158 unique variants, of which 112 were imputed (median imputation quality rsq = 0.92, range 0.31–1.00) and 46 were directly genotyped. Thirty-three variants were associated with eGFRcrea (Supplementary Data 1a), 44 with eGFRcys (Supplementary Data 1b), 11 with UACR (Supplementary Data 1c), 82 with urate (Supplementary Data 1d), and four with urea (Supplementary Data 1e). Over 60% (106/174) of these associations were not significant (*n* = 102) or could not be tested due to MAC < 5 (*n* = 4) when we performed a sensitivity ExWAS that used only the ~167 K samples with WES, which demonstrates the imputation-based increase in statistical power.

Of all 174 associations, 158 were phenotype-specific (Supplementary Data 1; Methods) and are described in the subsequent paragraphs. Between 30% (eGFRcys) and 62% (urate) of ExWAS variants mapped into GWAS loci (index SNP ± 500 kb; Methods). To assess whether any of the ExWAS results were driven by common (MAF > 1%) variants identified by previous GWAS of the respective trait, ExWAS were repeated while adjusting for the known common variants: effect estimates were largely unchanged as shown in Supplementary Fig. 3 (*R*^2^ estimates from regression analyses comparing unadjusted versus adjusted effect estimates were 0.99 for eGFRcrea, 0.97 for UACR, and 0.98 for urate; Methods), indicating that most of the rare variants tested in this ExWAS are statistically independent of known common GWAS variants. Notable exceptions were observed for urate, where effect sizes of rare variant associations in *SLC2A9*, the GWAS locus with the largest effect on urate^18^, were attenuated by more than 50% upon common variant adjustment.

### Rare variants associated with kidney function (eGFRcrea, eGFRcys, urea)

To distinguish variants that are likely related to biomarker metabolism from those that are truly relevant for kidney function, we assessed the overlap and direction of effects across the three kidney function markers eGFRcrea, eGFRcys, and urea (Supplementary Data 1). We prioritized 32 kidney function genes that showed (i) a significant association with one or both GFR estimates, (ii) direction-consistent effects, (iii) at least nominal significance (*p* < 0.05) for the respective other GFR estimate, and (iv) inverse associations with urea as well as with an ICD-10 based definition of CKD (Table 1; Supplementary Data 2; Methods). Thirteen (41%) of these prioritized genes are known to contain rare variants that cause monogenic diseases with a kidney phenotype: *CLDN10*, *CUBN*, *G6PC1*, *HNF4A*, *LRP2*, *NPHS1*, *PKD2*, *PKHD1*, *SLC12A1*, *SLC34A1*, *SLC34A3*, *SLC6A19*, and *SLC7A9*^19^. In fact, some of the identified variants are known pathogenic mutations for these conditions (e.g., p.Ser192Leu (rs199690076) for *SLC34A3*, p.Asp173Asn (rs121434346) for *SLC6A19*). Noteworthy candidates supported by kidney phenotypes in model organisms but not yet established as human kidney disease genes include *EPB41L5* and *FNIP1* (Table 1; Supplementary Data 2). In addition, several genes, such as *MITF*, have been linked to kidney cancers but not to reduced kidney function or CKD.

**Table 1:** Summary of evidence for a gene associated with kidney function

Gene symbol^a^	Kidney disease risk	ExWAS^b^	Gene burden^b^	Tissue/cell type expression^c^	Known GWAS locus^d^	Mouse with kidney phenotype
*ACSM2A*	down		X	PT	rs77924615	No
*ALDOB*	down	x	x	PT		no
*ARHGEF16*	down		x	EP; PE		yes
*CCNP*	down	x			rs59343080	no
*CGNL1*	up	x		EP; DVR	rs12148280	no
*CLDN10*^#^	up	x	x	EP	rs7326821	yes
*CLPX*	up	x	x	LOH		no
*CUBN*^#^	down	x		PT		yes
*EPB41L5*	up	x		POD		no
*ERBB4*	up	x		EP; CT	rs1851285	no
*FNIP1*	up	x	x			yes
*GATA5*	down	x		GE		yes
*G6PC1*^#^	down		x	PT		no
*HNF4A*^#^	up		x	PT	rs736820	yes
*LCN8*	up	x				no
*LRP2*^#^	down	x	x	PT	rs16823029	yes
*MITF*	up	x	x	LOH; CT	rs60551165	no
*NFAT5*	down	x		PC	rs113441031	yes
*NPHS1*^#^	down	x		POD	rs3814995	yes
*NRG4*	up		x	EP; PE	rs10851885	no
*PKD2*^#^	up	x		EP		yes
*PKHD1*^#^	down	x	x	EP; PC	rs12212034	yes
*RBM47*	up	x			rs166775	no
*RNF186*	up	x		PT		no
*SLC12A1*^#^	up		x	EP; LOH		yes
*SLC22A2*	up	x	x	PT	rs3119304	no
*SLC34A1*^#^	up		x	PT	rs10866705	yes
*SLC34A3*^#^	up	x	x	PT	rs28490558	yes
*SLC5A3*	up	x	x	EP	rs2834320	no
*SLC6A19*^#^	down	x	x	PT		yes
*SLC7A9*^#^	down	x		PT	rs8101667	yes
*VPS9D1*	down	x		DVR	rs154656	no

Genes associated with more than one kidney function measure (eGFRcreat, eGFRcys, urea) and direction-consistent association with CKD from ExWAS and gene-based tests are listed.

^a^“#” indicates that a monogenic kidney disease associated with this gene is reported in OMIM.

^b^“x” indicates the presence in a single variant ExWAS or association test that groups alleles in a gene (gene-level burden test).

^c^Kidney cell type in which the gene is specifically expressed. *PT* proximal tubule, *LOH* loop of Henle, *DVR*  descending vasa recta endothelium, *EP* epithelial progenitor, *PC* principal cell, *PE* pelvic epithelium, *POD* podocyte, *GE* glomerular endothelium, *CT* connecting tubule.

^d^dbSNP ids of variants of known eGFR GWAS loci based on Stancick et al.^28^ within ±100 kb of the variant position or gene start/end.

The value of investigating multiple kidney function markers was illustrated by the *SLC22A2* frameshift variant p.Phe24ThrfsTer4 (rs8177505), which was strongly associated with eGFRcrea (*p* = 1.9 × 10^−61^), but showed little or no association with eGFRcys (*p* = 4.1 × 10^−3^) or urea (*p* = 0.38). *SLC22A2* encodes an organic cation transporter of creatinine, among other substrates. It is, therefore, likely that this variant reflects transport-related changes in creatinine rather than a mechanism that results in reduced GFR.

**Fig. 2:**
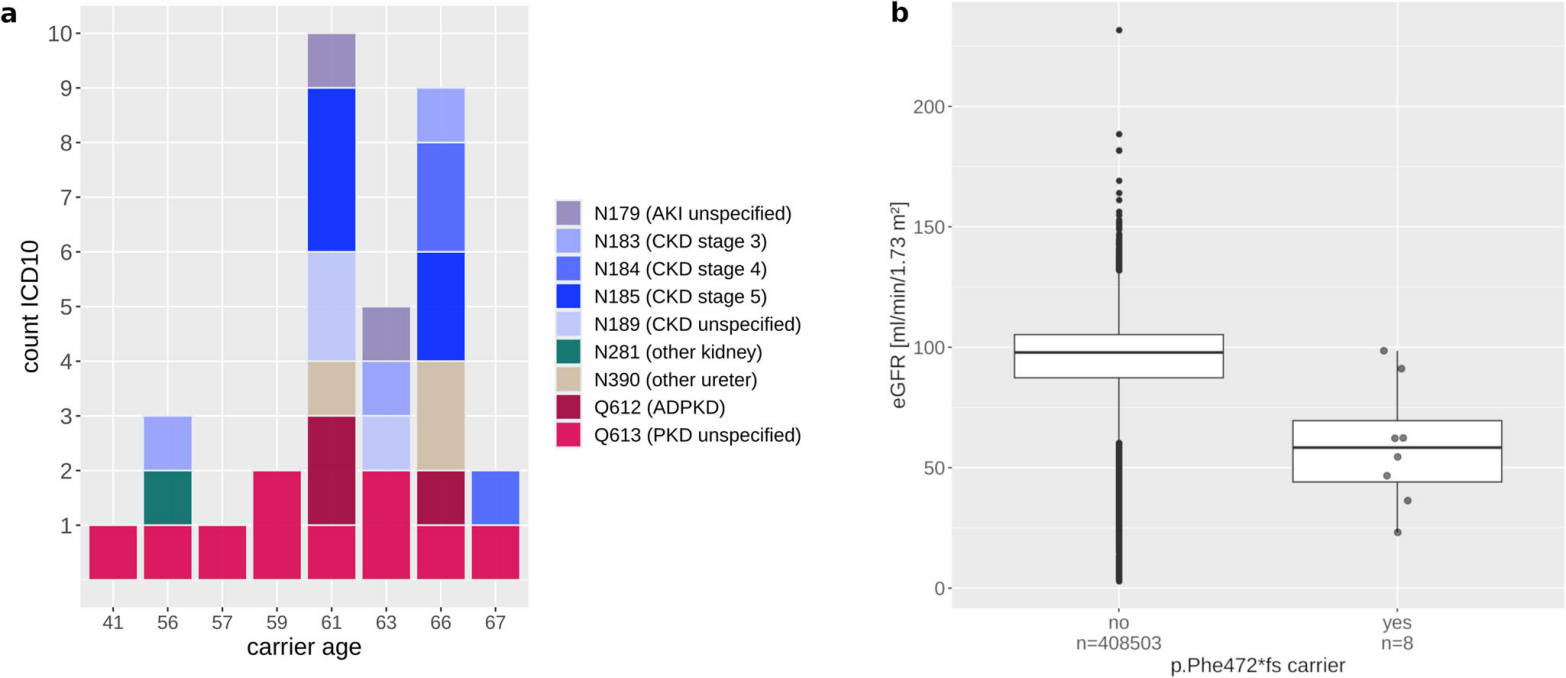
Details for carriers of the *PKD2* p.Phe472*fs variant. **a** Kidney ICD10 diagnosis by the carrier, color-coded (red—cystic kidney disease codes, blue—CKD codes). Carrier age was annotated in the columns. The median age was 60 (range 41–67). **b** Boxplot of eGFRcrea for the non-carriers of the p.Phe472*fs (4:88046737:TC:T) frameshift variant (left) and of the carriers (right); eGFRcrea of carriers ranged from 25 to 108 ml/min/1.73 m^2^ (mean 65, SD 28). The boxes represent the first to the third quartile, the horizontal line the median, and the whiskers extend to 1.5 times the interquartile range.

Of particular interest was a previously unreported variant, p.Phe472*fs (a one-base deletion chr4:88046737:TC:T) in *PKD2* with MAC = 8 (eGFRcys *p* = 5.8 × 10^−10^). *PKD2* is a major CKD gene whose mutations cause autosomal-dominant polycystic kidney disease (ADPKD), the most frequent monogenic kidney disease. The detected *PKD2* variant is absent from the GnomAD and ClinVar databases, but we found its association with CKD highly significant (OR > 1000, *p* = 3.7 × 10^−15^). The eight carriers in the UK Biobank were not closely related when considering kinship coefficient pairs of <0.0625 estimated genome-wide using common alleles. Upon inspection of inpatient hospital records (codes Q61.3 and/or Q61.2; Methods), we found that all eight carriers had clinical diagnoses of polycystic kidney disease (Fig. 2a), thereby validating the unbiased, imputation-powered association findings. Five of the carriers had CKD-related ICD codes, including one person whose kidney disease had progressed to kidney failure (Fig. 2a). eGFRcrea was clearly lower in carriers compared to non-carriers (Fig. 2b). The variant was directly sequenced in three carriers, and imputed in five (rsq = 0.99), demonstrating the effectiveness of the imputation approach and the value of combining complementary biomarker and ICD code-based information.

### Rare variants associated with kidney damage (UACR) and serum urate

The 11 ExWAS variants found associated with UACR map into the five genes *IGFLR1*, *COL4A3*, *COL4A4*, *CUBN*, and *NPHS1*. The high linkage disequilibrium (LD) between the variants in *IGFLR1* and *NPHS1* (Supplementary Data 1) suggests that the association with *IGFLR1* results from a shared haplotype with the neighboring *NPHS1*, a known cause of monogenic proteinuric kidney disease. Among the 82 urate-associated variants, 16 mapped into a region on chromosome 4 that includes *SLC2A9*, and another 30 variants mapped into an extended region on chromosome 11 that includes *SLC22A12*. Consistent with the physiology of the urate-reabsorption mediating transporters encoded by these genes, the urate-associated variants had a negative effect on urate levels and showed strong protection from gout. Known disease causing variants in genes associated with UACR and urate, as well as putative novel causative alleles, are described in detail in the Supplementary Results and Supplementary Fig. 4.

### Genes associated with kidney traits from aggregate variant testing

**Fig. 3:**
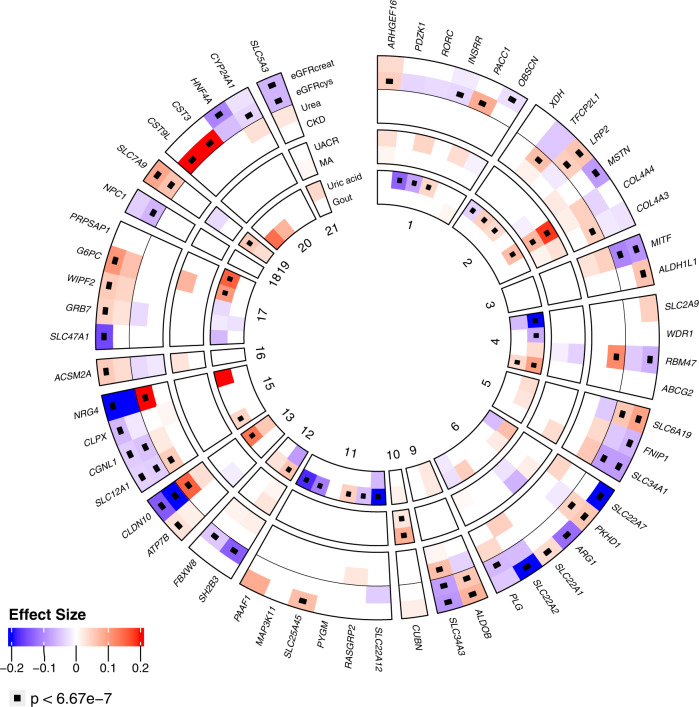
Associated genes across phenotypes. Circular heatmap for genes significantly associated with at least one phenotype of interest. Genes are depicted in the radials with one band per phenotype and divided by chromosome. Coloring according to effect size and direction. Significant gene-phenotype pairs (*p* < 6.8 × 10^−7^) are marked with a small black box. Effect size color is only shown for nominally significant (*p* < 0.05) gene-phenotype associations. Binary trait effect sizes are scaled by 10% (range: −2 to 2). Two-sided *p*-values were obtained from linear regression models of mask variant risk allele dosage on phenotypes.

We implemented gene-level burden tests for all five phenotypes over 18,727 genes to increase power to detect associations with rare variants of MAC ≥ 1 that may cluster in individual genes. Two alternative clustering methods (masks) were applied: (a) high confidence loss-of-function and missense variants, including protein truncating and other damaging variants (ptv_dmg, Methods) and (b) missense variants assumed to be deleterious by several in silico prediction tools including the CADD score (dmg_cadd). We identified 83 significant gene-trait associations for 57 unique genes (*p* < 6.7 × 10^−7^; Fig. 3, Table 2, Table 3, Supplementary Fig. 5, Methods). Thirty-one genes were associated with eGFRcrea, 22 with eGFRcys, 2 with UACR, 21 with urate, and 7 with urea (Supplementary Fig. 6). Together with the single variant results, associations were identified in a total of 105 genes. Detailed test statistics including the direction of effect for all phenotypes and masks are given in Supplementary Data 3. Effect directions were consistent with clinical expectations across related phenotypes, with the effects on eGFRcrea and eGFRcys being in the same direction, and the effects on eGFR and urea in opposite directions (Fig. 3).

**Table 2:** Overview of gene-based test results for eGFRcrea and eGFRcys

Chr	Position (b38)	Gene	eGFRcreatp-value	eGFRcys *p*-value	Urea *p*-value	UACR *p*-value	Urate *p*-value	Mask
*eGFRcrea*								
6	160171061	*SLC22A2*	**6.8 × 10**^**−81**^	1.7 × 10^−^^3^	3.8 × 10^−^^1^	6.3 × 10^−^^1^	5.2 × 10^−^^3^	ptv_dmg
6	43295694	*SLC22A7*	**3.0 × 10**^**−**^^**38**^	3.8 × 10^−^^1^	2.0 × 10^−^^1^	6.8 × 10^−^^1^	1.5 × 10^−^^5^	dmg_cadd
17	19495385	*SLC47A1*	**3.1 × 10**^**−**^^**34**^	1.9 × 10^−^^1^	1.1 × 10^−^^1^	8.9 × 10^−^^1^	2.2 × 10^−^^6^	dmg_cadd
2	169127109	*LRP2*	**2.0 × 10**^**−**^^**31**^	**8.3 × 10**^**−**^^**24**^	1.2 × 10^−^^3^	6.0 × 10^−^^1^	**7.1 × 10**^**−**^^**13**^	ptv_dmg
9	137230757	*SLC34A3*	**2.1 × 10**^**−**^^**30**^	**2.1 × 10**^**−**^^**27**^	**2.0 × 10**^**−**^^**14**^	5.3 × 10^−^^1^	3.8 × 10^−^^1^	dmg_cadd
5	1201595	*SLC6A19*	**1.6 × 10**^**−**^^**27**^	**3.8 × 10**^**−**^^**12**^	2.7 × 10^−^^6^	5.9 × 10^−^^1^	2.9 × 10^−^^5^	dmg_cadd
6	51615299	*PKHD1*	**1.1 × 10**^**−**^^**24**^	**4.0 × 10**^**−**^^**17**^	2.9 × 10^−^^3^	7.6 × 10^−^^3^	1.8 × 10^−^^4^	ptv_dmg
19	32830509	*SLC7A9*	**5.0 × 10**^**−**^^**23**^	**9.7 × 10**^**−**^^**12**^	1.6 × 10^−^^1^	3.2 × 10^−^^3^	**8.0 × 10**^**−**^^**10**^	dmg_cadd
3	126103562	*ALDH1L1*	**9.2 × 10**^**−**^^**16**^	5.0 × 10^−^^1^	7.0 × 10^−^^1^	4.0 × 10^−^^1^	6.3 × 10^−^^2^	dmg_cadd
15	65148219	*CLPX*	**2.2 × 10**^−^^**14**^	8.5 × 10^−^^3^	1.6 × 10^−^^1^	2.6 × 10^−^^1^	4.8 × 10^−^^1^	dmg_cadd
9	101420560	*ALDOB*	**4.6 × 10**^**−**^^**14**^	**1.4 × 10**^**−**^^**8**^	9.2 × 10^−^^4^	5.2 × 10^−^^2^	3.5 × 10^−^^2^	ptv_dmg
11	65375192	*SLC25A45*	**3.7 × 10**^**−**^^**11**^	4.7 × 10^−^^1^	7.7 × 10^−^^1^	5.8 × 10^−^^2^	1.8 × 10^−^^1^	dmg_cadd
6	131470832	*ARG1*	**4.5 × 10**^**−11**^	3.7 × 10^−^^1^	6.1 × 10^−^^1^	3.2 × 10^−^^3^	3.9 × 10^−^^1^	dmg_cadd
17	42900797	*G6PC*	**3.1 × 10**^**−9**^	3.7 × 10^−^^4^	3.4 × 10^−^^1^	2.8 × 10^−^^4^	**3.9 × 10**^**−**^^**9**^	dmg_cadd
5	131641714	*FNIP1*	**2.0 × 10**^**−**^^**8**^	2.8 × 10^−^^5^	1.8 × 10^−^^3^	1.3 × 10^−^^1^	1.5 × 10^−^^2^	dmg_cadd
17	39737927	*GRB7*	**3.6 × 10**^**−**^^**8**^	3.7 × 10^−^^4^	8.0 × 10^−^^4^	4.1 × 10^−^^1^	2.0 × 10^−^^2^	dmg_cadd
16	20451461	*ACSM2A*	**5.0 × 10**^**−**^^**8**^	1.5 × 10^−^^4^	3.5 × 10^−^^3^	1.3 × 10^−^^2^	4.9 × 10^−^^1^	ptv_dmg
2	190055700	*MSTN*	**6.4 × 10**^**−**^^**8**^	1.0	7.7 × 10^−^^1^	2.8 × 10^−^^1^	1.1 × 10^−^^1^	dmg_cadd
17	40219304	*WIPF2*	**7.7 × 10**^**−**^^**8**^	1.1 × 10^−^^5^	8.2 × 10^−^^1^	9.9 × 10^−^^1^	1.3 × 10^−^^3^	dmg_cadd
20	44355700	*HNF4A*	**3.2 × 10**^**−7**^	6.8 × 10^−^^3^	4.9 × 10^−^^1^	5.4 × 10^−^^1^	1.1 × 10^−^^1^	ptv_dmg
1	228208063	*OBSCN*	**5.0 × 10**^−^^**7**^	7.9 × 10^−^^2^	5.4 × 10^−^^1^	4.8 × 10^−^^2^	6.6 × 10^−^^1^	ptv_dmg
6	160121815	*SLC22A1*	**5.2 × 10**^−^^**7**^	3.4 × 10^−^^1^	1.7 × 10^−^^1^	8.2 × 10^−^^1^	1.6 × 10^−^^1^	dmg_cadd
13	51930436	*ATP7B*	**5.4 × 10**^−^^**7**^	4.8 × 10^−^^3^	7.5 × 10^−^^1^	1.9 × 10^−^^2^	8.8 × 10^−^^1^	ptv_dmg
*eGFRcys*								
20	23626706	*CST3*	6.0 × 10^−^^1^	**0.0**	9.6 × 10^−^^1^	9.9 × 10^−^^1^	9.9 × 10^−^^1^	ptv_dmg
12	111405923	*SH2B3*	2.6 × 10^−^^1^	**8.7 × 10**^**−**^^**43**^	8.6 × 10^−^^2^	2.2 × 10^−^^2^	**2.3 × 10**^**−**^^**9**^	dmg_cadd
13	95433604	*CLDN10*	**2.1 × 10**^−^^**8**^	**2.7 × 10**^**−**^^**17**^	**5.0 × 10**^−^^**8**^	2.7 × 10^−^^1^	**4.6 × 10**^**−**^^**8**^	dmg_cadd
15	57375967	*CGNL1*	**1.7 × 10**^**−**^^**10**^	**5.2 × 10**^**−**^^**15**^	3.4 × 10^−^^3^	8.8 × 10^−^^1^	6.0 × 10^−^^1^	dmg_cadd
3	69739435	*MITF*	**1.1 × 10**^**−**^^**10**^	**1.8 × 10**^**−**^^**13**^	3.0 × 10^−^^3^	6.1 × 10^−^^2^	6.6 × 10^−^^1^	ptv_dmg
5	177379235	*SLC34A1*	**8.6 × 10**^**−**^^**12**^	**4.2 × 10**^**−**^^**12**^	3.6 × 10^−^^5^	1.3 × 10^−^^2^	3.0 × 10^−^^1^	ptv_dmg
6	160702238	*PLG*	2.5 × 10^−^^6^	**6.6 × 10**^**−**^^**10**^	4.4 × 10^−^^4^	7.6 × 10^−^^1^	7.2 × 10^−^^3^	ptv_dmg
21	34073578	*SLC5A3*	**2.7 × 10**^**−**^^**9**^	**2.3 × 10**^**−**^^**9**^	3.8 × 10^−^^2^	2.9 × 10^−^^1^	1.5 × 10^−^^4^	ptv_dmg
20	23564732	*CST9L*	1.9 × 10^−^^1^	**2.9 × 10**^**−**^^**9**^	5.3 × 10^−^^1^	4.5 × 10^−^^1^	5.4 × 10^−^^1^	ptv_dmg
1	3454665	*ARHGEF16*	3.2 × 10^−^^5^	**1.8 × 10**^**−**^^**8**^	9.3 × 10^−^^2^	8.6 × 10^−^^4^	5.6 × 10^−^^1^	dmg_cadd
20	54153446	*CYP24A1*	1.4 × 10^−^^4^	**3.1 × 10**^**−**^^**8**^	7.4 × 10^−^^6^	8.7 × 10^−^^1^	5.5 × 10^−^^2^	dmg_cadd
1	212363931	*PACC1*	4.4 × 10^−^^1^	**4.8 × 10**^**−**^^**8**^	4.0 × 10^−^^1^	4.9 × 10^−^^2^	2.1 × 10^−^^1^	ptv_dmg
12	116910950	*FBXW8*	1.8 × 10^−^^1^	**3.2 × 10**^**−7**^	2.7 × 10^−^^1^	5.0 × 10^−^^1^	1.3 × 10^−^^3^	dmg_cadd
18	23506184	*NPC1*	1.2 × 10^−^^3^	**3.4 × 10**^**−7**^	9.8 × 10^−^^1^	7.1 × 10^−^^1^	6.0 × 10^−^^1^	ptv_dmg

Includes genes are associated with at least one phenotype (*p* < 6.67  ×  10^−^^7^). Significant gene-phenotype associations are marked in boldface. Two-sided p-values were obtained from linear regression models of mask variant risk allele dosage on phenotypes. Genes are grouped by the phenotype with the lowest association *p*-value. *p*-Value is given for the most significant mask. *Chr* chromosome, *b38* genomic build 38, *eGFR* estimated glomerular filtration rate, *UACR* urinary albumin-to-creatinine ratio, *ptv_dmg* high confidence loss of function variants including protein truncating and other damaging variants, *dmg_cadd* missense variants that are also assumed to be deleterious by several in silico prediction tools including the CADD score (Methods).

**Table 3:** Overview of gene-based test results for Urea, UACR, and Urate

Chr	Position (b38)	Gene	eGFRcreatp-value	eGFRcys *p*-value	Urea *p*-value	UACR *p*-value	Urate *p*-value	Mask
*Urea*								
4	40423267	*RBM47*	**1.4 × 10**^**−**^^**10**^	1.1 × 10^−^^5^	**5.7 × 10**^**−**^^**24**^	1.4 × 10^−^^3^	2.5 × 10^−^^4^	dmg_cadd
2	227164624	*COL4A3*	4.1 × 10^−^^4^	5.9 × 10^−^^3^	**1.8 × 10**^**−**^^**10**^	4.0 × 10^−^^5^	1.9 × 10^−^^1^	ptv_dmg
2	121216587	*TFCP2L1*	2.4 × 10^−^^6^	1.6 × 10^−^^6^	**8.2 × 10**^**−**^^**10**^	4.7 × 10^−^^1^	**6.4 × 10**^**−**^^**7**^	dmg_cadd
15	48178438	*SLC12A1*	**6.2 × 10**^**−**^^**7**^	**6.4 × 10**^**−**^^**8**^	**2.3 × 10**^**−**^^**9**^	7.7 × 10^−^^1^	**3.8 × 10**^**−**^^**8**^	dmg_cadd
15	75935969	*NRG4*	**9.5 × 10**^**−**^^**8**^	6.1 × 10^−^^4^	**1.1 × 10**^**−**^^**8**^	5.2 × 10^−^^2^	1.5 × 10^−^^4^	ptv_dmg
*UACR*								
10	16823966	*CUBN*	2.7 × 10^−^^5^	7.5 × 10^−^^1^	1.3 × 10^−^^1^	**1.3 × 10**^**−**^^**63**^	7.3 × 10^−^^4^	dmg_cadd
2	227002714	*COL4A4*	8.1 × 10^−^^1^	7.7 × 10^−^^3^	1.6 × 10^−^^3^	**1.1 × 10**^**−**^^**30**^	**6.5 × 10**^**−**^^**7**^	ptv_dmg
*Urate*								
11	64590641	*SLC22A12*	2.6 × 10^−^^1^	1.5 × 10^−^^3^	1.4 × 10^−^^1^	2.3 × 10^−^^1^	**0.0**	dmg_cadd
4	9771153	*SLC2A9*	4.2 × 10^−^^3^	4.2 × 10^−^^1^	8.3 × 10^−^^1^	1.7 × 10^−^^1^	**8.3 × 10**^**−**^^**114**^	dmg_cadd
4	88090150	*ABCG2*	1.3 × 10^−^^1^	1.3 × 10^−^^1^	4.9 × 10^−^^1^	4.0 × 10^−^^1^	**7.1 × 10**^**−**^^**39**^	dmg_cadd
1	145670852	*PDZK1*	8.3 × 10^−^^2^	5.7 × 10^−^^3^	4.3 × 10^−^^1^	7.5 × 10^−^^2^	**4.2 × 10**^**−**^^**27**^	dmg_cadd
11	64746389	*PYGM*	5.3 × 10^−^^1^	5.0 × 10^−^^1^	5.1 × 10^−^^1^	7.2 × 10^−^^1^	**2.6 × 10**^**−**^^**16**^	dmg_cadd
17	76309478	*PRPSAP1*	7.1 × 10^−^^1^	7.3 × 10^−^^1^	9.7 × 10^−^^1^	6.4 × 10^−^^1^	**3.8 × 10**^**−**^^**14**^	dmg_cadd
11	65597756	*MAP3K11*	6.0 × 10^−^^1^	5.2 × 10^−^^1^	7.3 × 10^−^^1^	4.2 × 10^−^^1^	**2.3 × 10**^**−**^^**13**^	dmg_cadd
2	31334321	*XDH*	5.2 × 10^−^^1^	2.5 × 10-1	8.4 × 10^−^^1^	3.6 × 10^−^^1^	**2.0 × 10**^**−**^^**11**^	dmg_cadd
1	156840063	*INSRR*	7.6 × 10^−^^3^	**1.6 × 10**^**−**^^**7**^	6.2 × 10^−^^2^	4.0 × 10^−^^1^	**5.5 × 10**^**−**^^**10**^	dmg_cadd
4	10074339	*WDR1*	6.7 × 10^−^^1^	3.6 × 10^−^^1^	2.4 × 10^−^^1^	2.4 × 10^−^^1^	**3.5 × 10**^**−**^^**9**^	dmg_cadd
1	151806071	*RORC*	1.4 × 10^−^^1^	3.8 × 10^−^^2^	1.4 × 10^−^^1^	1.2 × 10^−^^2^	**1.1 × 10**^**−**^^**7**^	ptv_dmg
11	73876699	*PAAF1*	1.6 × 10^−^^2^	5.2 × 10^−^^1^	5.0 × 10^−^^1^	8.8 × 10^−^^1^	**2.7 × 10**^**−**^^**7**^	ptv_dmg
11	64726911	*RASGRP2*	6.4 × 10^−^^1^	1.5 × 10^−^^1^	4.5 × 10^−^^2^	1.3 × 10^−^^1^	**5.2 × 10**^**−**^^**7**^	dmg_cadd

Includes genes are associated with at least one phenotype (*p* < 6.67  ×  10^−^^7^). Significant gene-phenotype associations are marked in boldface. Two-sided *p*-values were obtained from linear regression models of mask variant risk allele dosage on phenotypes. Genes are grouped by the phenotype with the lowest associatithe on *p*-value. *p*-Value is given for the most significant mask. *Chr* chromosome, *b38* genomic build 38, *eGFR* estimated glomerular filtration rate, *UACR* urinary albumin-to-creatinine ratio, *ptv_dmg* high confidence loss of function variants, including protein truncating and other damaging variants, *dmg_cadd* missense variants that are also assumed to be deleterious by several in silico prediction tools including the CADD score (Methods).

All variants contributing to any of the identified gene-trait pairs are provided in Supplementary Data 4, allowing for the identification of variant consequence (e.g., truncating) and putative mechanism of action (gain- vs. loss-of-function). To assess whether genes were identified as a result of aggregation of multiple variants or mostly driven by the contribution of a single variant, we examined and categorized the 57 unique genes into three groups, using the trait for which the gene achieved the smallest *p*-value (Supplementary Fig. 7, Methods): (1) “multi-variant signal”, where ≥two variants were needed to achieve significance (*n* = 16; example: *SLC34A1* with eGFRcys); (2) “multi-variant signal with one variant sufficient to achieve significance” (*n* = 34; example: *SLC7A9* with eGFRcrea); (3) “not a multi-variant signal” (*n* = 7; example: *SLC25A45* and eGFRcrea).

### Genes associated with kidney function (eGFRcrea, eGFRcys, urea)

We compiled a list of 32 genes that were significantly associated with eGFRcrea or eGFRcys either via GBT or ExWAS analyses, and which showed a direction-consistent and at least nominally significant (*p* < 0.05) association with the respective other GFR estimate, along with a direction consistent association with urea and CKD (Table 1; Supplementary Data 2). Thirteen of the 32 genes (41%) are listed in the OMIM catalog as genes that, when mutated, can cause monogenic diseases of the kidney, 19 (59%) mapped into a known eGFR GWAS locus, and 16 (50%) showed a corresponding kidney phenotype when genetically manipulated in mice. Of note, seven of the 32 genes (22%) were not identified by ExWAS, but only through aggregate variant testing.

There were two genes that have not yet been described as causative for monogenic kidney diseases in humans, but for which genetically manipulated mice show a kidney disease phenotype, namely *ARHGEF16* and *FNIP1*. Little is known about Rho Guanine Nucleotide Exchange Factor 16, encoded by *ARHGEF16*. Phenotypic characterization of genetically manipulated mice in the Mouse Genome Informatics resource showed enlarged kidneys and abnormal kidney morphology (MGI: 2446219). *Fnip1/Fnip2* double knockout mice develop polycystic kidneys and renal cancer^20^, while Fnip1 disruption is sufficient for renal cyst formation^21^

### Genes associated with kidney damage (UACR) and serum urate

The two genes identified through aggregate variant testing as associated with UACR, *CUBN* (*p* = 1.3 × 10^−63^) and *COL4A4* (*p* = 1.1 × 10^−30^), have well-known roles in monogenic diseases that feature proteinuria^22,23^ (Table 1; Supplementary Data 2).

For serum urate, aggregate variant testing identified significant associations with 21 genes, six of which were not identified through ExWAS for this trait (*SLC7A9*, *CLDN10*, *XDH*, *INSRR*, *RORC*, and *PAAF1*; Table 3). *XDH* encodes for xanthine dehydrogenase, a central enzyme in purine metabolism, of which uric acid is the end-product. Rare mutations are a known cause of autosomal recessive xanthinuria type I (MIM #278300): affected individuals show very low serum urate levels. Accordingly, we observed a negative association with serum urate (beta = −0.04, *p* = 2.0 × 10^−11^) and a protective effect on gout (gout OR = 0.8, beta = −0.22, *p* = 0.16), consistent with the effectiveness of XDH inhibitors for urate-lowering treatment. The association with *PAAF1, e*ncoding a protein involved in proteasome assembly regulation, and serum urate has not been reported previously. Interestingly, *PAAF1* was described as an interactor of *ALDH16A1*, another locus identified here^24^. The exceptionally large effects of rare, damaging variants in *SLC22A12* and *SLC2A9*, two genes encoding for the major transporters responsible for urate reabsorption in the kidney, on serum urate and gout have been described in previous WES studies of serum urate^10^ and were confirmed in this study.

### Validation of imputation-based signals using sequencing data and comparison with published UK Biobank WES study

**Fig. 4:**
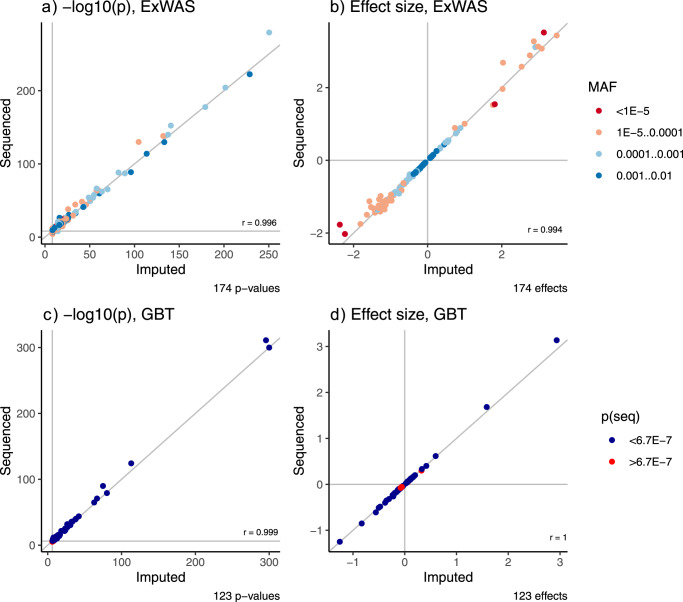
Validation of imputation-based signals using data from whole exome sequencing of all corresponding UK Biobank participants. Scatter plots of −log_10_-transformed *p*-values (**a**) and effect sizes (**b**) for single variant (ExWAS) and −log_10_-transformed *p*-values (**c**) and effect sizes (**d**) for the gene-based test (GBT) analyses comparing association statistics from partially imputed (*x*-axis) and fully sequenced (*y*-axis) data. Single variant results are color-coded by minor allele frequency (MAF), and GBT results are color-coded by *p*-value (blue—*p*-value below the significance threshold of 6.7 × 10^−7^, red—above threshold). *r* denotes the Pearson correlation coefficient. For panel a, two-sided *p*-values were obtained from linear mixed effect models (REGENIE) of effect allele dosage on phenotypes. For panel **c**, two-sided *p*-values were obtained from linear regression models of mask variant risk allele dosage on phenotypes.

Over the course of this study, WES data from the full UK Biobank became available. We therefore validated all discovered significant ExWAS and GBT associations by repeating the respective analysis using sequence rather than imputed information for the same set of 408,511 individuals (Methods). For the ExWAS associations, we observed excellent concordance of the association *p*-values between imputed and sequenced variants (Pearson correlation coefficient of 0.996, Fig. 4a). Statistical significance was reached for 159/174 sequenced variants (Supplementary Data 1). The 15 variants that did not pass the significance threshold showed association *p*-values closely above the threshold (median: 2.5 × 10^−8^, IQR: 1.0 × 10^−8^−6.1 × 10^−8^), and the association effect sizes of imputed compared to sequenced variants were highly correlated across the range of minor allele frequencies (Pearson coefficient 0.994, Fig. 4b). Secondly, we also observed excellent agreement of association p-values originating from the 123 significant GBT associations (Pearson correlation 0.999, Fig. 4c). We found that 112/123 genes were statistically significantly associated. Again, variants that did not reach statistical significance had *p*-values closely above the threshold (median: 1.4 × 10^−6^; IQR: 1.0 × 10^−8^−2.3 × 10^−6^), and the correlation coefficient of the GBT effect estimates obtained from imputed and from fully sequenced data was 0.999 (Fig. 4d, Supplementary Data 3).

Next, we performed a systematic comparison of our results to those from the largest phenome-wide WES study that also analyzed kidney-related biomarkers in the UK Biobank^14^. We revealed an overlap of 55 genes using the statistical significance definition of our study (Methods). Our approach identified 50 additional genes, while 39 of the genes identified by Backman and colleagues were not present in our main results (Supplementary Results, Supplementary Fig. 8). More detailed investigations of genes specific to one or the other study revealed that the majority of them was also nominally significant in the respective other studies.

### Kidney-related genes are highly expressed in specific kidney cell types

**Fig. 5:**
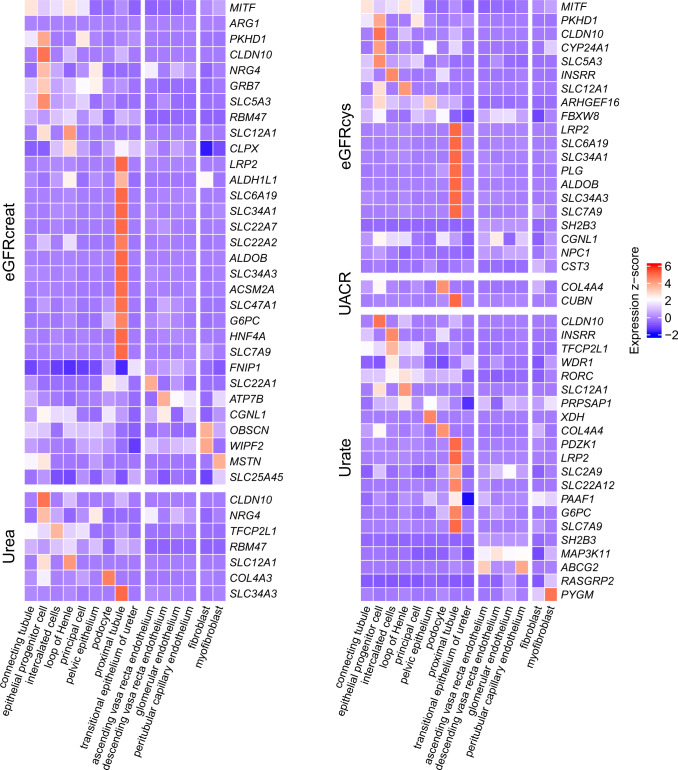
Expression of associated genes from gene-level analyses in kidney cell types. Heatmaps showing cell type-specific expression of associated genes in eGFRcrea, eGFRcys, urea, UACR, and urate. The expression *z*-score values are based on single-cell RNA-seq data from Stewart et al.^25^ Fifteen non-immune kidney cell types are grouped into nephron, endothelium, and stroma, with 9, 4, and 2 cell types each. Genes in each heatmap are ordered by the maximum expression *z*-score along the 15 cell types.

The expression of genes identified through gene-based tests was assessed at single-cell resolution using publicly available single-cell RNA-seq data from human mature kidney cells^25^. Earlier studies^26,27^ have reported that human monogenic and complex kidney disease genes are expressed primarily in a single kidney cell type. Comparing expression levels across kidney cell types confirmed this conclusion also for the genes identified in our project (Fig. 5). The majority of genes related to eGFRcrea, eGFRcys, and serum urate, were highly expressed in the proximal tubule, followed by epithelial progenitor cells for eGFR-related genes. Cell type-specific expression of genes associated with UACR reflected the known pathophysiology of the respective gene: *COL4A3* and *COL4A4* were found to be expressed specifically in podocytes, consistent with their role in the glomerular filter, and *CUBN* in the proximal tubule, consistent with its role in the tubular reabsorption of filtered albumin.

### Kidney-related genes are associated with additional clinical biomarkers and diseases

**Fig. 6:**
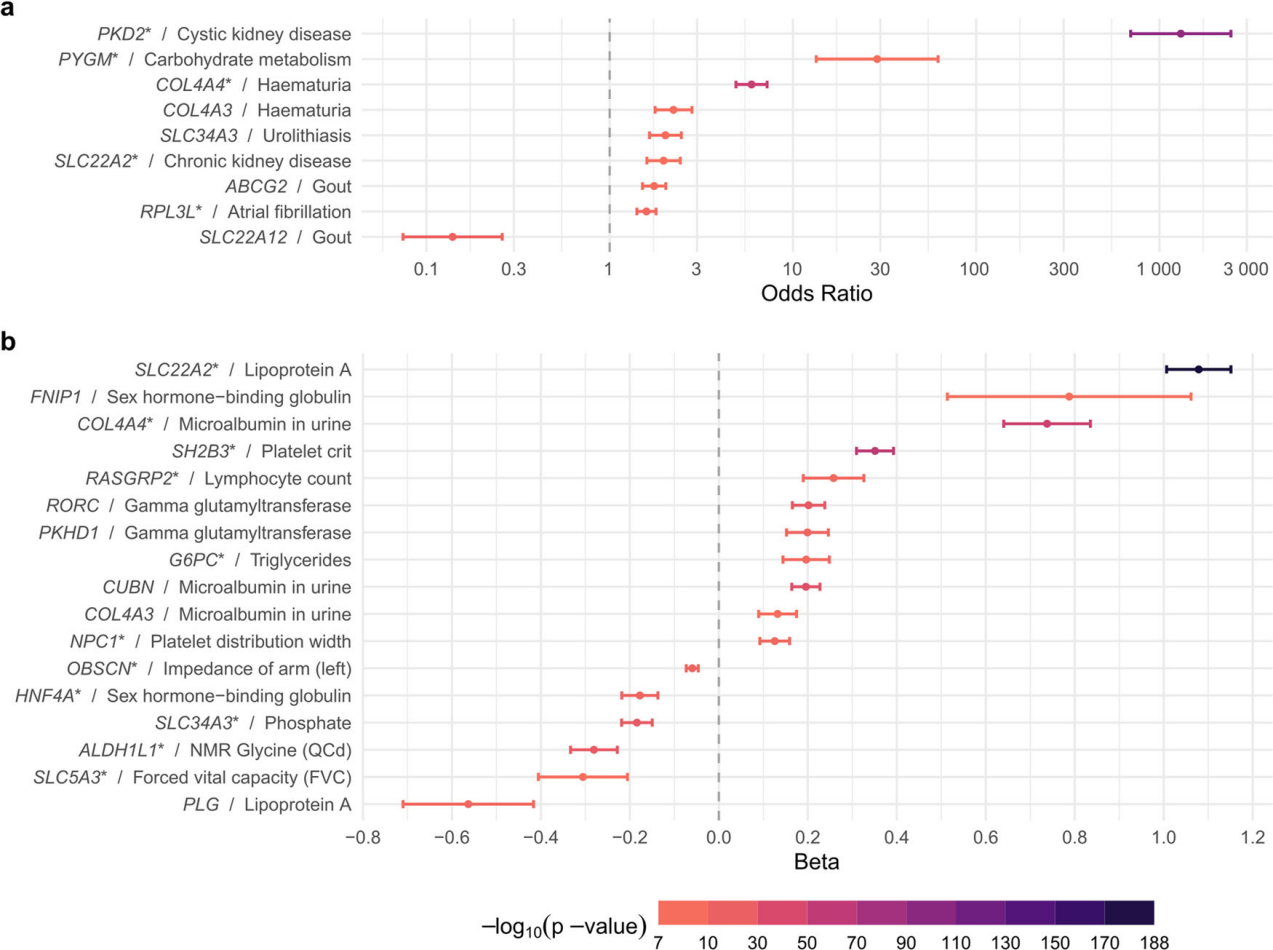
Phenome-wide association study for kidney genes. Phenome-wide association study of genes identified in our single variant ExWAS or gene-based study with other phenotypes in the UK Biobank. For every gene, the odds ratio or beta of the UK Biobank phenotype with the smallest *p*-value of a gene-based association test is displayed. Genes with multiple significant phenotype associations are marked with an asterisk (*). Full results, including numbers of biologically independent samples for all shown UK phenotypes, are available in Supplementary Data 5. Only UK Biobank associations with *p*-value < 5 × 10^−8^ (as reported by the respective studies), and more than 5 cases (for binary traits) or more than 5 individuals (for quantitative traits) were considered. **a** Odd ratios are shown as center points with error bars representing 95% confidence intervals for binary traits. **b** Betas are shown as center points with error bars representing 95% confidence intervals for quantitative traits, resulting from a linear regression model correcting for age, sex, and age × sex.

We next examined whether the genes we identified in association with the five kidney traits were associated with clinical biomarkers and diseases using publicly available association results from the UK Biobank^15^ (Methods). This can identify associations with non-kidney traits and diseases that may go unnoticed when only studying individual patients in clinical genetic studies. Across the phenome, there were numerous instances where the assumed loss-of-function of the evaluated genes resulted in increased disease risk or changes in continuous markers of disease (Fig. 6, Supplementary Data 5). Often, these associations reflected known clinical signs and symptoms observed among patients with the resulting monogenic diseases. For example, carriers of rare damaging variants in both *COL4A3* and *COL4A4* showed higher odds of hematuria, urinary symptoms and kidney problems, with ORs as high as 6 for *COL4A4* and hematuria (Fig. 6a). *COL4A4* variant carriers also showed higher odds of hypertensive disease, consistent with nephritic syndrome commonly observed in Alport’s syndrome patients (MIM #203780), and lower hemoglobin concentrations and hematocrit percentage, consistent with hematuria (Fig. 6b). Similarly, carriers of rare variants in *SLC34A3* showed lower phosphate levels and higher odds of urolithiasis (Fig. 6a), consistent with phenotypes of patients affected by hypophosphatemic rickets with hypercalciuria due to recessive *SLC34A3* mutations (MIM #241530).

### Enrichment analyses highlight biologically plausible pathways

We performed Gene Ontology (GO) enrichment analyses to identify biological processes, molecular functions, and cellular components that were significantly enriched for genes identified by our WES analysis and compared the results to those obtained when using genes identified by published GWAS^4,18,28^ of the corresponding trait as input. To ensure sufficient power, the analyses were carried out for eGFR and for serum urate, where WES identified >5 genes. Pathways that were significantly enriched in both WES-based as well as GWAS-based analyses contained many examples that are highly plausible (Supplementary Data 6). For instance, pathways related to eGFR contained kidney/renal system/urogenital development as well as those related to transport processes across the apical and basolateral plasma membrane (Supplementary Fig. 9A), whereas implicated pathways for serum urate contained “urate metabolic process” as well as those related to the brush border membrane (Supplementary Fig. 9B), where the most important urate transport proteins that greatly influence serum urate levels are expressed.

### Experimental validation of a previously unreported eGFR-associated *CLDN10* frameshift allele

While previously unreported, putatively pathogenic variants such as the frameshift variant in *PKD2* can be validated through orthogonal evidence from hospital diagnostic codes, experimental validation remains the gold-standard to establish variant pathogenicity. As proof of principle, we, therefore, performed experimental studies of a previously unreported eGFR-associated frameshift allele in *CLDN10*, encoding the tight junction protein claudin-10. This allele was detected in 32 heterozygous carriers in the imputed WES dataset and significantly associated with its lead trait eGFRcys (carriers had 0.99 standard deviations lower transformed eGFRcys values compared to non-carriers, *p* = 4.2 × 10^−12^) as well as with other kidney-related traits. The corresponding results from the directly sequenced 167 K UK Biobank participants available at the outset of this project were 13 carriers, 0.96 standard deviations, and a non-significant *p* = 8.3 × 10^−5^.

Isoform claudin-10a is expressed in the kidney proximal tubule and the uterus, claudin-10b in the thick ascending limb of Henle’s loop, and various exocrine glands^29–31^. Mutations in *CLDN10* can cause the autosomal recessively inherited HELIX syndrome^32^, which features hypohidrosis, electrolyte imbalance, lacrimal gland dysfunction, ichthyosis, and xerostomia. Abnormal eGFR was found for some mutations^33–36^, but not for others^35–38^. In our study, the monoallelic deletion (p.Asp223GlufsTer34, 13:95577994:GAT:G, Supplementary Fig. 10) was associated with lower eGFRcys, higher urea, and higher odds of CKD. The resulting frameshift (fs) causes loss of the C-terminal pdz-binding motif needed for binding of the tight junction protein claudin-10 to the scaffolding protein ZO-1^39^. A previously described single nucleotide deletion (13:95577980 del) in two Saudi Arabian families (claudin-10 fs(SA))^37^ also caused pdz-binding motif loss and resulted in HELIX syndrome in homozygous carriers. Heterozygous family members were unaffected.

**Fig. 7:**
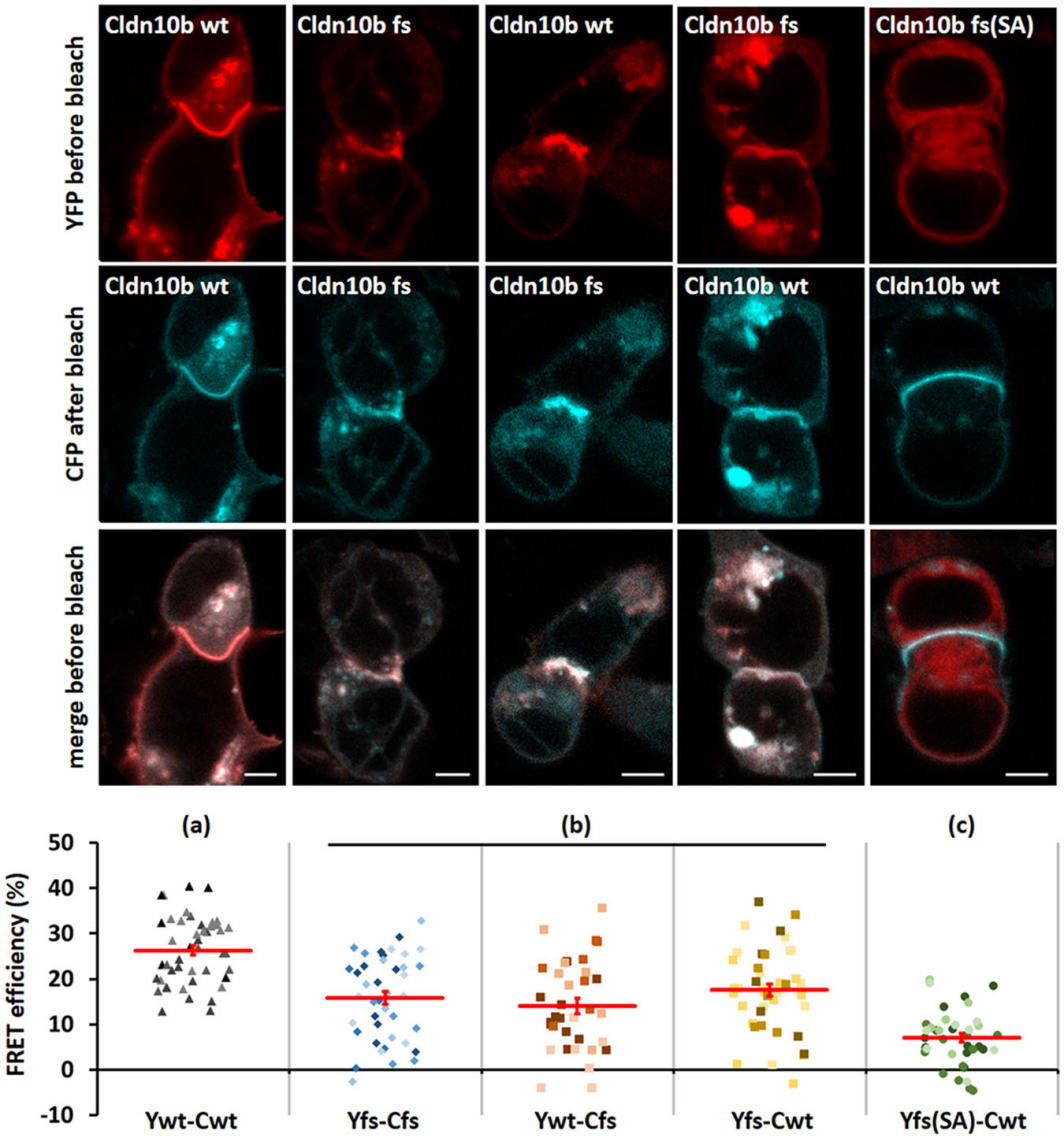
Claudin-10b wt and fs: subcellular distribution and FRET efficiency. *Upper panels*: co-transfection of YFP (red) and CFP(cyan)-tagged Cldn10b wt, fs, or fs(SA), respectively. The majority of wt–wt contacts were long and had a smooth appearance, whereas the majority of fs–fs contacts were short, and had an interrupted (dashed or ‘ragged’) appearance, likely due to claudin-10b within vesicles close to the plasma membrane rather than truly contact-enriched claudin-10b. Cldn10b wt co-localized with claudin-10b fs in cell–cell contacts (merge); however, these contacts had an appearance similar to the contacts observed in cells expressing only Cldn10b fs. As previously described (Alzahrani et al., 2021), Cldn10b fs(SA) did not insert into the plasma membrane but was retained in intracellular compartments (endoplasmic reticulum). The co-expressed Cldn10b wt was unaffected by the presence of Cldn10b fs(SA). Cell–cell contacts had a wt-like appearance. Bars: 5 µm. *Lower panel*: FRET efficiency as an indicator for *cis*-interaction was highest when only Cldn10b wt was present (**a**, gray triangles, *n* = 46 from *m* = 5 independent transfections). When only Cldn10b fs (blue diamonds, *n* = 41, *m* = 5) was present, or when Cldn10b fs was combined with Cldn10b wt (**b**; squares; YFP-wt–CFP-fs, red symbols, *n* = 34, YFP-fs–CFP-wt, yellow symbols *m* = 4; *n* = 43, *m* = 4), FRET was highly significantly lower (**a** vs. **b**, 3.96E−12). When Cldn10b fs(SA) was combined with Cldn10b wt (**c**; green circles, *n* = 38, *m* = 4), FRET efficiency was highly significantly lower than FRET efficiencies observed under all other conditions (**a** vs. **c**: *p* = 2.16E−08; **b** vs. **c**: *p* < 1E−16). ANOVA and Tukey Posthoc test. Different transfections per condition are indicated by different shades of the symbols. Red lines indicate mean FRET efficiency ± SEM.

Overexpression of fluorescent protein-claudin-10b fusion proteins in HEK 293 cells showed that, similar to claudin-10b wildtype (wt), claudin-10b fs (but not claudin-10b fs(SA)) was able to enrich in contacts between two transfected cells, indicating the formation of tight junction-like structures and suggesting wt-like trans-interaction of claudin-10b fs (Fig. 7; Supplementary Fig. 11). However, claudin-10b fs enrichments had an impaired appearance (predominantly smooth in wt, irregular in fs), that was not corrected by the co-transfection of claudin-10b wt and was mirrored by reduced FRET efficiency of claudin-10b fs within these contacts as a measure of claudin *cis* interaction (Fig. 7). Whereas claudin-10b fs(SA) neither interacted with claudin-10 wt nor affected its localization, claudin-10b fs interfered with claudin-10 wt localization, likely explaining a mild dominant effect that is distinct from the molecular phenotype of the HELIX syndrome causing mutation. These findings underscore the value of experimental studies for the characterization of alleles that do not result in typical monogenic disease presentations, to yield a more complete picture of the allelic spectrum. Imputation-powered studies of the coding genome, such as our study, can identify and prioritize such variants for the required further experimental investigations.

## Discussion

We performed ExWAS and gene-level analyses of five measures of kidney function and disease in up to 408,513 white British participants of the UK Biobank study. To increase statistical power, genotypes for 241,620 individuals with available chip genotypes but no sequencing data at the time of our study were imputed for 7,575,566 variants using the available WES data. We sought to characterize our findings across different kidney phenotypes, cell types, and tissues; and to explore the relationship of identified genes across the phenome. We identified associations at 158 unique single variants and at 57 genes with gene-level tests, all at or driven by rare variants. We validated these imputation-based associations using recently released exome sequences from the full set of UK Biobank participants.

Our study complements previous phenome-wide ExWAS screens in the UK Biobank^13,40^ by being the first dedicated effort to assess associations of kidney function and disease in this dataset. Whereas previous broad screens included kidney-relevant biomarkers such as creatinine and cystatin C as two out of thousands of phenotypes, we used these biomarkers to estimate the most common clinical measure of kidney function, the GFR, compared and contrasted associations across different kidney function markers, and validated results by examining ICD-10 and biomarker-based binary phenotypes and kidney diseases. We were thereby able to distinguish genes that are likely involved in biomarker metabolism from those truly related to reduced kidney function or kidney damage. In addition, we showed that most rare variant associations were independent of common variant associations in nearby GWAS loci. Moreover, we extended a previously introduced WES imputation approach^16^ to show that a three-times larger reference panel improves the imputation quality further and facilitates the discovery of many additional significant associations. This has implications for future studies, that may use the now available WES data from the full UK Biobank to impute additional study samples.

In our study, the detection of numerous genes and variants known to cause monogenic kidney diseases when mutated underscore the validity of the results and emphasize that imputation-powered studies of the exome are a feasible and attractive option when whole exome sequencing can only be financed in a subset of the study sample. This holds true even for imputed variants with a MAC < 10, as highlighted by the rare *PKD2* variant p.Phe472*fs that we detected in association with eGFR. Although this variant is absent from public databases, all carriers had ICD codes related to cystic kidney disease. Thus, the variant can be considered a novel disease-causing mutation that was uncovered by an imputation-powered association study of the coding genome.

As proof of principle, we experimentally established that a previously unreported eGFR-associated frameshift variant in *CLDN10* is a functional allele. The monoallelic deletion causes loss of the C-terminal pdz-binding motif needed for binding of the tight junction protein claudin-10 to the scaffolding protein ZO-1. The claudin-10b frameshift protein interfered with the localization of the wild type, which is likely explaining the mild dominant-negative effect.

In terms of genetic architecture of the studied kidney function markers, there was substantial overlap between genes identified in association with eGFRcrea, eGFRcys, and urea, as would be expected for these complementary filtration markers. Conversely, none of the genes associated with UACR and only a few of the genes associated with serum urate were also associated with the filtration markers. This observation supports the separate study of the genetic architecture of GFR-related traits^4^, UACR^9^, and serum urate^8,18^, as is current practice for common variant studies.

The findings of this study have to be seen in the light of some limitations. To maximize imputation quality by increasing the sample size of the reference panel, we focused on samples of white British ancestry only, which limits the generalizability of our results. The presented associations have been chosen after rigorous statistical analysis; however, we did not replicate them in an independent external data set. Finally, imputation quality depends highly on how often an allele is observed in the reference panel, which makes it impossible to impute variants private to samples not in the reference panel, as observed for *PKD1*, and could lead to poor imputation results for ultra-rare variants such as singletons or doubletons.

In summary, we identified 105 unique genes and characterized 174 rare variant-associations and 83 gene-associations for five measures of kidney function and disease in a WES-based imputed data set of the UK Biobank and made these results publicly available using PheWeb. These findings revealed genetic determinants of kidney function and will help to direct future functional and clinical studies.

## Methods

### Sample selection

Analogous to other studies^16,17^, 408,511 participants that were part of the white British ancestry subset (mean age 48.9 ± 8, 54% female) according to the genotype quality control of the UK Biobank were included in this study. Informed consent was obtained by the UK Biobank for all participants.

### Phenotype definition

We considered five kidney-related phenotypes: eGFRcrea (based on creatinine, UKB field 30,700, instance 0), eGFRcys (based on cystatin C, UKB field 30720, instance 0), UACR (UKB fields 30,500 and 30,510), serum urate (UKB field 30,880, instance 0), and urea (UKB field 30,670, instance 0). eGFRcrea and eGFRcys were calculated using the CKD-EPI equations^41^ and winsorized to 15 and 200 ml/min/1.73 m^2^. To calculate UACR, values for urinary albumin below the detection limit were set to the detection limit value. All phenotypes were inverse-normal transformed.

We fitted linear regression models to the phenotypes, adjusting for sex, age, and the first 40 genetic principal components, as provided by the UK Biobank. For secondary analyses, the same models were additionally adjusted for 639 SNPs for eGFR^28^, 63 SNPs for UACR^9^, and 184 SNPs for urate^18^ to account for the potential effect of common variants.

CKD and gout were defined using ICD10 codes from hospital inpatient records (N18.*, M10.*, UKB field 41270). Microalbuminuria was defined as UACR > 30 mg/g. ExWAS were carried out for these clinically relevant outcomes and used to annotate the findings for continuous kidney markers with respect to the direction and significance of their association with disease. To further characterize the risk allele carriers of selected trait-associated variants, kidney disease was additionally defined by ICD codes for acute kidney injury (N17.9), CKD (N18.3, N18.4, N18.5, N18.9), polycystic kidney disease (Q61.2, Q61.3), and another kidney (N28.1) or ureter (N39.0) disease. Information on allopurinol treatment was obtained from a verbal interview on medication usage.

### Genotype imputation

First, genotyped variants (hap_v2) of all 408,511 individuals of White British ancestry (WBA) were lifted to GRCh38 to match the genotype build of the WES calls. To generate the reference panel for the within-cohort imputation of the WES variants into the remainder of the cohort, autosomal WES variants with MAC > 1 of all 166,891 samples of WBA were merged with genotyped variants with MAC > 1 on the same set of individuals. For the target scaffold, directly genotyped variants with a MAF > 0.0001 in the remainder of the WBA cohort (*n* = 241,620) were selected. Both panels were split into matching chunks of roughly 30,000 variants with 1 Mb overlap, generating a total of 324 chunks. Each chunk was phased with Eagle2 v2.4.1 (reference panel: --Kpbwt=50000 --pbwtIters=5; target scaffold: --Kpbwt=100000 --pbwtIters=5 --pbwtOnly)^42^. Imputation was performed with minimac4 on each chunk^43^. Imputed chunks were concatenated phase-aware per chromosome by cutting off 0.5 Mb of each end. In total, 7,596,602 variants that have not been directly genotyped were imputed in the 241,620 genotyped individuals. The imputed calls were merged with the sequenced calls, keeping only sites with maximum missingness of 0.05 and estimated imputation accuracy rsq > 0.3, resulting in 7,479,293 variants in 408,511 individuals.

For validation purposes, the imputation was repeated once, as described above, randomly removing 10,000 individuals from the reference panel. Imputation quality was assessed in these individuals by comparing the correlation of imputed dosages as well as hard calls with the genotypes derived from the WES, excluding directly genotyped sites, which resulted in *n* = 2,191,400 variants for validation. To assess imputation accuracy also for the very rare variants, the validation set was divided into tranches by MAC and MAF of the variants in the reference panel, with one tranche per MAC up to 9, followed by larger tranches for low-frequency and common variants. To enable a comparison to the imputation quality achieved by Barton et al. on a reference panel of ~50 K^16^, the exact same MAF range was used. Imputation quality statistics restricting to different classes of variants (SNPs or indels) or different levels of missingness for rare and common variants are available in Supplementary Fig. 12 and Supplementary Data 7.

### Functional annotation

Variant annotation was performed using the Ensembl Variant Effect Predictor (VEP)^44^ version 101 with standard settings, which comprises annotations of the canonical transcripts, gene symbols, and frequencies from the Genome aggregation database (gnomAD v2.1). VEP plugins were used to add the REVEL score (v2020-5)^45^ and the CADD score (v3.0)^46^. Loss-of-function variants were rated using the LoFtee VEP plugin (version 2020-8)^47^ and classified as high-confidence or low-confidence. The in-silico scores LRT (likelihood-ratio test for deleteriousness), M.CAP (Mendelian Clinically Applicable Pathogenicity Score), MetaSVM (meta-score incorporating multiple in-silico scores)^48^ and FathMM.XF (Prediction score of pathogenic point mutations)^49^ were added using the dbNSFP (v4.1a)^50^.

### Significance threshold definition

For the single variant analysis, variants in canonical transcripts and HGNC genes with MAC ≥ 5, MAF < 1%, imputation info score ≥0.5, estimated imputation quality rsq ≥ 0.3, VEP impact ≥LOW and VEP consequence not “synonymous” were considered (n = 1,844,188). Quantile-quantile (QQ) and Manhattan plots with different filter settings were assessed. Because of the strong between-phenotype correlation (Pearson correlation coefficient between eGFRcrea and eGFRcys = 0.61; Supplementary Fig. 13), Bonferroni multiple-testing correction was applied by accounting for 4 rather than 5 phenotypes, and the significance threshold was set at *p* = 0.05/4/1844188 = 6.78 × 10^−^^9^. Similarly, for the gene level analysis, where 18,727 genes were tested, the significance threshold was set at 0.05/18727/4 = 6.67 × 10^−^^7^.

### Single variant analysis

A single variant, whole-exome linear mixed model association analyses were performed with the REGENIE software package^51^ v2.0.2 in two steps:

*REGENIE Step 1*: Whole-genome regression model using the recommended parameter setting. The UK Biobank DNA microarray genotypes (hap_v2) were lifted to build GRCh38 and filtered using the recommended settings (genotype call rate > 0.1; Hardy–Weinberg equilibrium *p*-value < 1e−15; MAC > 100; MAF > 0.01; sample call rate > 0.90).

*REGENIE Step 2*: Association analysis was performed on imputed dosage levels (BGEN v1.2 8-bit data format) for 408,511 individuals and 1,844,188 variants. LD was estimated afterward between all pairs of significantly associated variants using PLINK v1.90b2^52^. We restricted the association analysis to variants with MAC ≥ 5, as recommended by the REGENIE developers^51^.

To calculate association statistics independent of common variants, we extracted 423, 63, and 114 independent, common SNPs previously reported to be associated with eGFR^4,28^, UACR^9^, and urate^18^, respectively, from the imputed UK Biobank data, set. We calculated residuals by regressing eGFR, UACR, and urate on all SNPs for a given trait in one regression analysis, and used these residuals as phenotypes in another ExWAS. Adjusted and unadjusted association results were compared with respect to their effect size estimates. Reported *R*^2^ measures originated from a linear regression model.

The PheWeb software (https://github.com/statgen/pheweb) with default settings was used to create a local PheWeb instance that displays the results of the single variant analysis and is available under https://ckdgen-ukbb.gm.eurac.edu/.

### Gene-level analysis

Two masks (ptv_dmg, dmg_cadd) that defined variants to be aggregated per gene were created based on the UKBB OQFE exome map using the VEP annotations described above. For ptv_dmg, variants were required to pass the following criteria: LoFtee high-confidence loss-of-function, or (missense consequence and MetaSVM score > 0), or FathMM_XF_coding score > 0.5. Using these settings, the ptv_dmg filter included 15,710 genes with a median of 30 variants per gene (interquartile range, IQR 13–68). For dmg_cadd, variants were included with (the consequence “missense” (or worse), a CADD score > 20), a REVEL score > 0.5, or a M_CAP rating “deleterious”. This resulted in 18,727 genes with a median of 114 variants per gene (IQR 54–203).

The gene-level analysis was performed with the SeqMeta R package (seqMeta: An R package for meta-analyzing region-based tests of rare DNA variants; A Voorman, J Brody, H Chen, T Lumley, B Davis – v1.6.7, 2013). As genotype input, imputed hard call genotypes filtered for MAF < 0.01 and estimated imputation quality rsq ≥ 0.3 were used.

To determine how the variants aggregated by the masks for the respective gene contribute to the association signal of the gene test, variants were ordered by their single variant ExWAS p-value from lowest to highest (*n* variants). Then, two metrics were defined: (1) add-one-in: for *i* from 1 to *n*, the burden test was computed using only the *i* variants with the lowest single variant p-value; (2) leave-one-out: for *i* from 0 to *n* *−* 1, the burden test was computed, removing the *i* variants with the lowest single variant p-values. Let p_1_, …, p_*i*_,…p_*n*_ be the p-values of the burden tests for the “add-one-in” tests. We call a gene-trait association a “multi-variant signal” if SD{−log_10_(p_*1*_),.., -log_10_(p_*n*_)} ≥ 0.5, that is, the p-values of the burden tests with different variant sets show some variance. To facilitate interpretation, we assign these associations into three categories. Category 1 associations comprise multi-variant signals, where at least two variants are needed to reach significance (p_1_ ≥ 6.7 × 10^−^^7^, see above). Multi-variant associations where one variant is sufficient to reach significance (p_1_ < 6.7 × 10^−^^7^) are assigned to category 2. Associations that are not multi-variant signals were assigned to category 3.

### Validation of imputation-based signals using data from whole exome sequencing of all corresponding UK Biobank participants

ExWAS were repeated for the 174 significant (*p* < 6.8 × 10^−9^) associations as well as the analyses for significant (*p* < 6.7 × 10^−7^) associations derived from GBT of aggregated variants, using sequence information for the same set of 408,511 individuals for which imputed sequences were available. Genotype calls were extracted from the final UKBB population-level OQFE exome variants (field: 23,158, version: Jul 2022) using PLINK; association analyses were performed with the same phenotype files and the same software packages as described above.

### Cell type-specific expression of the associated genes in the kidney

To examine the expression of the associated genes in human kidney cell types, a single-cell RNA-seq dataset was downloaded from https://www.kidneycellatlas.org/^25^. The dataset included expression profiling of 40,268 human mature kidney cells in 27 cell clusters. The dataset was processed using the R package Seurat v3^53^. The averaging expression values of cells in the same cluster were calculated and then transformed to *z*-scores so that an expression matrix of genes and cell clusters was obtained. We then used the Bioconductor package ComplexHeatmap v2.9.3 to generate heatmaps of the associated genes for each phenotype based on the expression matrix^54^. Immune cells were not included in the heatmaps.

### GO-term enrichment analyses

We performed GO enrichment analyses using Bioconductor packages clusterProfiler v3.18.1^55^. The input genes were from our WES analyses as well as those from published GWAS^4,18,28^. The EnrichGO function of clusterProfiler was used. GO terms were considered significantly enriched if the FDR-adjusted *p*-value < 0.05.

### Experimental studies on a previously unreported *CLDN10* frameshift mutation

#### Generation of claudin-10 mutant construct

The CLDN10b frameshift (fs) mutant was generated by 2-step PCR, using a previously published human CLDN10b wildtype (wt) construct^29^ and human kidney cDNA as templates. The following custom-made primers (Eurofins Genomics, Ebersberg, Germany) were employed: FOR1a 5′-aaaggatccAatggctagcacggc-3′; REV1a 5′-ctcttttagacataagcatttttcaaactgttttgaaggg-3′; FOR2b 5′-cccttcaaaacagtttgaaaaatgcttatgtctaaaagag-3′; REV1b 5′-ttgaggaatattctcagattgcccccatg-3′; REV2 5′-TTTgatatcttatgggagggccttgatgggatc-3′. The final PCR product was cloned into pcDNA 3.1-based vectors containing the sequence for enhanced yellow (EYFP) or cyan (ECFP) fluorescent protein, using BamHI and EcoRV restriction sites, to obtain N-terminally tagged claudin fusion proteins.

### Culture and transfection of human embryonic kidney (HEK) 293 cells

HEK293 cells (CRL-1573, A.T.C.C., Manassas, VA, USA) were grown in 25 cm^2^ culture flasks in MEM-Earle’s media (Sigma-Aldrich, Munich, Germany) supplemented with 10% (v/v) fetal bovine serum, 100 U/mL penicillin, and 100 µg/ml streptomycin (Sigma-Aldrich), at 37 °C in a humidified 5% CO_2_ atmosphere. Cell line identity was confirmed by PCR single locus technology (Promega, PowerPlex 21 PCR Kit; Eurofins Medigenomix Forensik GmbH, Ebersberg, Germany) in 2018.

For transient transfection, HEK293 cells were seeded on poly-L-lysine (Sigma–Aldrich) coated coverslips in 6-well plates, at a density of 600,000 cells per well. 24 h later, cells were transfected by adding 500 µl cell culture medium (without supplements) per well, containing 10 µl polyethylenimine solution (Sigma-Aldrich, stock concentration 1 µg/µl), 2 µg EYFP- and 2 µg ECFP-plasmid DNA. Approximately 48 h after transfection, HEK293 cells were used for confocal laser scanning microscopy.

The following plasmid combinations were used: EYFP-Cldn10b wt–ECFP-Cldn10b wt, EYFP-Cldn10b fs–ECFP-Cldn10b fs, EYFP-Cldn10b wt–ECFP-Cldn10b fs, EYFP-Cldn10b fs–ECFP- Cldn10b wt. For comparison, the frameshift mutant previously found in Saudi Arabian HELIX syndrome patients^37^ was also included: EYFP-Cldn10b fs(SA)–ECFP-Cldn10b wt.

### Live cell imaging: Förster resonance energy transfer (FRET) and enrichment assays

Live cell imaging was employed to quantify *cis* (FRET assay, Supplementary Fig. 14) and trans (enrichment assay, Supplementary Fig. 15) interaction of claudins^56,57^. Cover slips with transfected HEK293 cells were transferred into a HEPES-buffered, HCO_3_^−^-free bath solution (in mM: 134.6 NaCl, 2.4 Na_2_HPO_4_, 0.6 NaH_2_PO4, 5.4 KCl, 1.2 CaCl_2_, 1 MgSO_4_, 10 HEPES, and 10 d(+)-glucose, pH 7.4) and placed in a heated microscope chamber (37 °C) of a Zeiss LSM 780 confocal microscope. ECFP and EYFP fluorescence was excited at 458 nm and 514 nm, and emission was detected at 470–500 nm and 530–600 nm, respectively.

For the enrichment assay, fluorescence intensity was determined at cell–cell contacts of two claudin-expressing cells (*I*_c_) and at the contacts of these two cells with cells not expressing claudins (*I*_m1_, *I*_m2_). The enrichment factor, EnF, was calculated as *I*_c_/(*I*_m1_ + *I*_m2_), as depicted in Supplementary Fig. 15, and considered positive if the value was >1.

For the FRET assay, YFP was bleached 3 times 15 pulses of the 514 nm laser line at 100% intensity, and the relative change in CFP fluorescence intensity before (*I*_b_) and after (*I*_a_) acceptor bleach was evaluated (FRET efficiency = (*I*_a_ − *I*_b_)/*I*_a_ × 100%), as depicted in Supplementary Fig. 14^57^. Ratios of YFP fluorescence intensity before bleach and CFP fluorescence intensity after bleach (YFP/CFP ratio) were calculated to assess the comparability of obtained FRET efficiencies.

### Reporting summary

Further information on research design is available in the Nature Portfolio Reporting Summary linked to this article.

## Supplementary information

Supplementary Information

Description of Additional Supplementary Files

Supplementary Data 1 to 7

Reporting Summary

## Data Availability

We used publicly available individual-level genotype and phenotype data from the UK Biobank (https://biobank.ndph.ox.ac.uk/showcase/). Access to these data needs to be requested from the UK Biobank. Our results are shared with the community using a comprehensive online resource: https://ckdgen-ukbb.gm.eurac.edu/ This work was conducted within approved UK Biobank application numbers 20272 and 64806.

## Code availability

Custom scripts and code are available online at https://github.com/genepi-freiburg/seqmeta (10.5281/zenodo.7564424). Code for other central tools is available online (REGENIE: https://rgcgithub.github.io/regenie, SeqMeta: https://rdrr.io/cran/seqMeta/, PLINK: https://www.cog-genomics.org/plink/).
