# Supplementary material for: Imputation-powered whole-exome analysis identifies genes associated with kidney function and disease in the UK Biobank
Source: Nat Commun. 2023 Mar 9;14:1287. doi: 10.1038/s41467-023-36864-8 (PMC9995463; doi:10.1038/s41467-023-36864-8)
Supplement: Supplementary file 1 — Supplementary Information [file 41467_2023_36864_MOESM1_ESM.pdf]

Imputation-powered whole-exome analysis identifies rare coding variants and genes associated with kidney function and disease in the UK Biobank

Supplementary Information

Matthias Wuttke*[1,2], Eva König*[3], Maria-Alexandra Katsara[1], Holger Kirsten[4,5], Saeed Khomeijani Farahani[6], Alexander Teumer[7,8], Yong Li[1], Martin Lang[3], Burulca Göcmen[1], Cristian Pattaro[3], Dorothee Günzel [6], Anna Köttgen**[1,9], Christian Fuchsberger**[3]

1 Institute of Genetic Epidemiology, Faculty of Medicine and Medical Center - University of Freiburg, Freiburg, Germany
2 Renal Division, Department of Medicine, Faculty of Medicine and Medical Center - University of Freiburg, Freiburg, Germany
3 Eurac Research, Institute for Biomedicine (affiliated to the University of Lübeck), Bolzano, Italy
4 Institute for Medical Informatics, Statistics and Epidemiology, University of Leipzig, Leipzig, Germany
5 LIFE Research Centre for Civilization Diseases, University of Leipzig, Leipzig, Germany
6 Clinical Physiology/Nutritional Medicine, Charité - Universitätsmedizin Berlin, Berlin, Germany 7 Institute for Community Medicine, University Medicine Greifswald, Greifswald, Germany
8 DZHK (German Center for Cardiovascular Research), Partner Site Greifswald, Greifswald, Germany
9 Department of Epidemiology, Johns Hopkins Bloomberg School of Public Health, Baltimore (Maryland), USA

* These authors contributed equally. ** These authors jointly supervised this work.

<u>Contents</u>

Contents 2
Supplementary Results 3
Gene-based association analysis of *PKD1* 3
Rare variants associated with kidney damage (UACR) 3
Rare variants associated with serum urate 4
Supplementary Figures 6
Supplementary Figure 1: Quantile-quantile plots for ExWAS analyses 6
Supplementary Figure 2: Manhattan plots for ExWAS analyses 7
Supplementary Figure 3: Identified associations with rare variants are largely independent from associations with common variants 8
Supplementary Figure 4. Distribution of UACR values by carrier genotype for variants in *CUBN* 9
Supplementary Figure 5: Quantile-quantile plots of gene-based test results 10
Supplementary Figure 6: Overlap plot between genes identified through gene-based testing with genes that when mutated can cause known monogenic kidney genes 12
Supplementary Figure 7: Add-one-in (blue) and leave-one-out (orange) plots for 56 significant gene – trait associations 13
Supplementary Figure 8: Details of the gene-based analyses for *PKD1* 17
Supplementary Figure 9: Pathway enrichment analyses 18
Supplementary Figure 10: Claudin-10b wildtype and frame-shift sequences. 19
Supplementary Figure 11: Trans-interaction of claudin-10b wt and fs in HEK 293 cell-cell contacts 20
Supplementary Figure 12: Imputation quality in 10,000 validation samples and 2,191,400 variants for different filtering thresholds 21
Supplementary Figure 13: Phenotype correlation 22
Supplementary Figure 14: FRET assay to quantify cis-interaction of tight junction proteins 23
Supplementary Figure 15: Enrichment assay to quantify trans-interaction of tight junction proteins. 24
Supplementary References 25

Supplementary Results

Gene-based association analysis of *PKD1*

The major kidney disease gene *PKD1* only showed nominally significant association with eGFR in our study (p=0.028, dmg_cadd, Supplementary Fig. 9). Further investigation of the genetic architecture of this gene in the WES source data used for phasing showed that most variants which would qualify for testing were singleton missense variants. Since variants with MAC=1 were excluded from the imputation reference panel, genes with a genetic architecture like *PKD1* are best detected from the original sequence data. However, the fact that we detected most of the creatinine-associated genes underscores that instances like *PKD1*, which are difficult to detect in imputation-powered gene-based tests, represent an exception.

Rare variants associated with kidney damage (UACR)

The 11 UACR-associated ExWAS variants map into the five genes *IGFLR1*, *COL4A3*, *COL4A4*, *CUBN*, and *NPHS1*. The latter four of the genes harbor variants causing monogenic kidney diseases featuring proteinuria, hematuria or both. Autosomal-dominant forms of Alport syndrome have been described for *COL4A3* and *COL4A4*.[1] For p.Gly695Arg (rs200287952) and p.Gly818Arg (rs868002181) in *COL4A3,* there are reports of individuals exhibiting a kidney phenotype consistent with Alport syndrome,[2,3] which, when manifesting with isolated proteinuria and hematuria, can easily be misdiagnosed as focal segmental glomerulosclerosis (FSGS).[4] One of the two variants in *COL4A4*, rs35138315, is classified as pathogenic for autosomal-recessive Alport syndrome by ClinVar. The second variant, a small in-frame deletion p.Ile29_Leu30del (rs771943519, $p=5.8\times10^{-9}$) is absent from GnomAD, but falls in a region with good sequencing (31.5x) coverage. It has been reported to be pathogenic in *trans* with another mutation in *COL4A4*.[5] We did not identify individuals homozygous for the variant alleles in *COL4A3* and *COL4A4*.

The rare *NPHS1* variant p.Ser937Asn (rs201194276) lies 10kb upstream but independent ($r^2=0$ and $D'=0$) of the known eGFR GWAS variant rs3814995 (MAF=26%).[6] p.Ser937Asn was additionally identified to be associated with serum creatinine in a previous UK Biobank ExWAS[7] and with UACR in another ExWAS study.[8] Rare damaging variants in

NPHS1 are a known cause of congenital Nephrotic Syndrome of the Finnish type, a disease featuring highly elevated UACR levels, implicating p.Ser937Asn as a likely causal allele.

Furthermore, we found 18 individuals homozygous for either p.Ala1690Val (rs141640975) or p.Asn2157Asp (rs144360241) in *CUBN*, which encodes the tubular re-uptake protein cubilin. Mutations in *CUBN* are associated with the autosomal recessive Imerslund-Gräsbeck syndrome characterized by vitamin B12 malabsorption and proteinuria but normal kidney function. *CUBN* variant carriers showed UACR values in a range that is typical for glomerular disease, highlighting the substantial amount of filtered albumin that is normally reabsorbed through cubilin-mediated endocytosis in the proximal tubule cells. The seven homozygous carriers of rs141640975 in *CUBN* had >12-fold higher UACR compared to non-carries (129 vs. 10 mg/g, two-sided Wilcox test $p=7\times10^{-6}$, **Supplementary Fig. 4**). We identified an additional *CUBN* variant, p.Gly1928Val (rs201513648), which was absent from ClinVar and GnomAD, but predicted as pathogenic, and thus may represent a new causal variant (**Supplementary Table 1**).

Lastly, p-values for associations of these variants with microalbuminuria (defined as "UACR >30 mg/g") were similar to the quantitative UACR associations.

Rare variants associated with serum urate

Among the 82 urate-associated variants, 16 mapped into a region on chromosome 4 that includes *SLC2A9*, and another 30 variants mapped into an extended region on chromosome 11 that includes *SLC22A12*. These genes encode GLUT9 and URAT1, respectively, the major urate transporters responsible for tubular reabsorption of filtered urate from the urine into the blood. Loss of function of these genes are known causes of monogenic renal hypouricemia type 2 (MIM #612076, *SLC2A9*) and type 1 (MIM #220150, *SLC22A12*). These observations were supported both by the almost exclusively negative effect of the corresponding ExWAS variants on urate levels and by the detection of known pathogenic variants such as p.Leu75Arg (rs863225072) in *SLC2A9* (**Supplementary Table 1d**). Further supporting these findings, urate-associated ExWAS variants showed strong protection from gout, a clinical condition resulting from elevated urate levels. In particular, rare variants in *SLC22A12* showed gout odds ratios (OR) as low as 0.2, and >2 SD lower urate levels from the

mean (**Supplementary Table 1d**). Both *SLC2A9* and *SLC22A12* have been identified in GWAS[9–11] and WES studies[12] of urate conducted in general population samples.

Other urate-associated variants also mapped into genes known to encode urate transporters, namely ABCG2 and OAT4 (*SLC22A11*). As opposed to GLUT9 and URAT1, ABCG2 and OAT4 have a role in urate secretion, and presumed loss of function variants identified in our screen were associated with higher urate levels and odds of gout. Particularly the p.Ala528Thr variant (rs45605536) in *ABCG2* showed strong association with gout (OR=2.2, p=4.2×10^{-4}). Other noteworthy associations included variants in *PYGM*, *ALDH16A1* and *PRPSAP1*, all of which encode for enzymes. Glycogen Phosphorylase, Muscle Associated (PYGM) is involved in glycogenolysis. Rare *PYGM* mutations cause the autosomal recessive McArdle disease (MIM #232600), a glycogen storage disorder, often featuring hyperuricemia and gout.[13] We identified the p.Arg50Ter stop-gain variant (rs116987552), recognized as pathogenic in ClinVar and here associated with gout (OR=1.3, p=0.046). *ALDH16A1* encodes a member of the aldehyde dehydrogenase family. The low frequency variant p.Pro527Arg (rs150414818) identified in our project was strongly associated with urate (p=2.0×10^{-58}), which translated to an almost 20-fold higher chance of gout (OR=18.6, p=2.3×10^{-21}; **Supplementary Table 1d**). This odds ratio is substantially larger than that reported in a previous rare variant association study from Iceland for the same variant (gout OR=3.1).[14] Variant carriers were more likely to be diagnosed with gout compared to non-carriers (7.8% vs. 1.8%, p<2.2×10^{-16}). Lastly, additional significant variants were identified in genes classically implicated in kidney diseases such as C*OL4A4* (rs35138315, a pathogenic stop-gain variant causing Alport Syndrome) and *LRP2* (rs34355135, **Table 2**). Overall, 51/82 (62%) urate-associated ExWAS variants mapped to known GWAS loci, with a substantial fraction attributed to *SLC2A9* and *SLC22A12*.

Supplementary Figures

Supplementary Figure 1: Quantile-quantile plots for ExWAS analyses

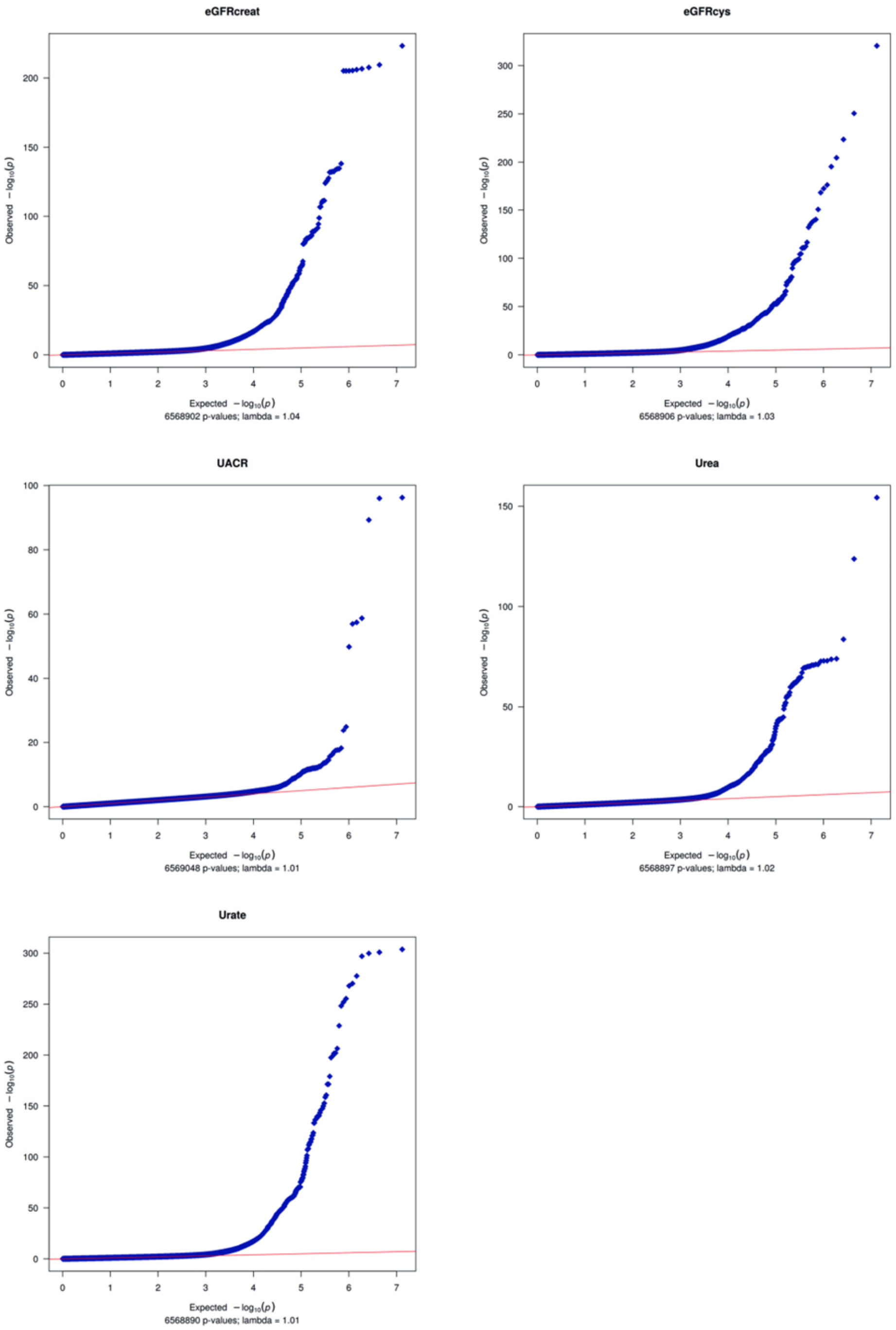

Plots of observed vs. expected association p-values for the five main phenotypes on a –log10 scale for all variants with at least one non-reference call (minor allele frequency (MAF) >0). The figure caption shows the number of tested variants and the inflation factor lambda per phenotype. Two-sided p-values were obtained from linear mixed effect models (REGENIE) of effect allele dosage on phenotypes.

Supplementary Figure 2: Manhattan plots for ExWAS analyses

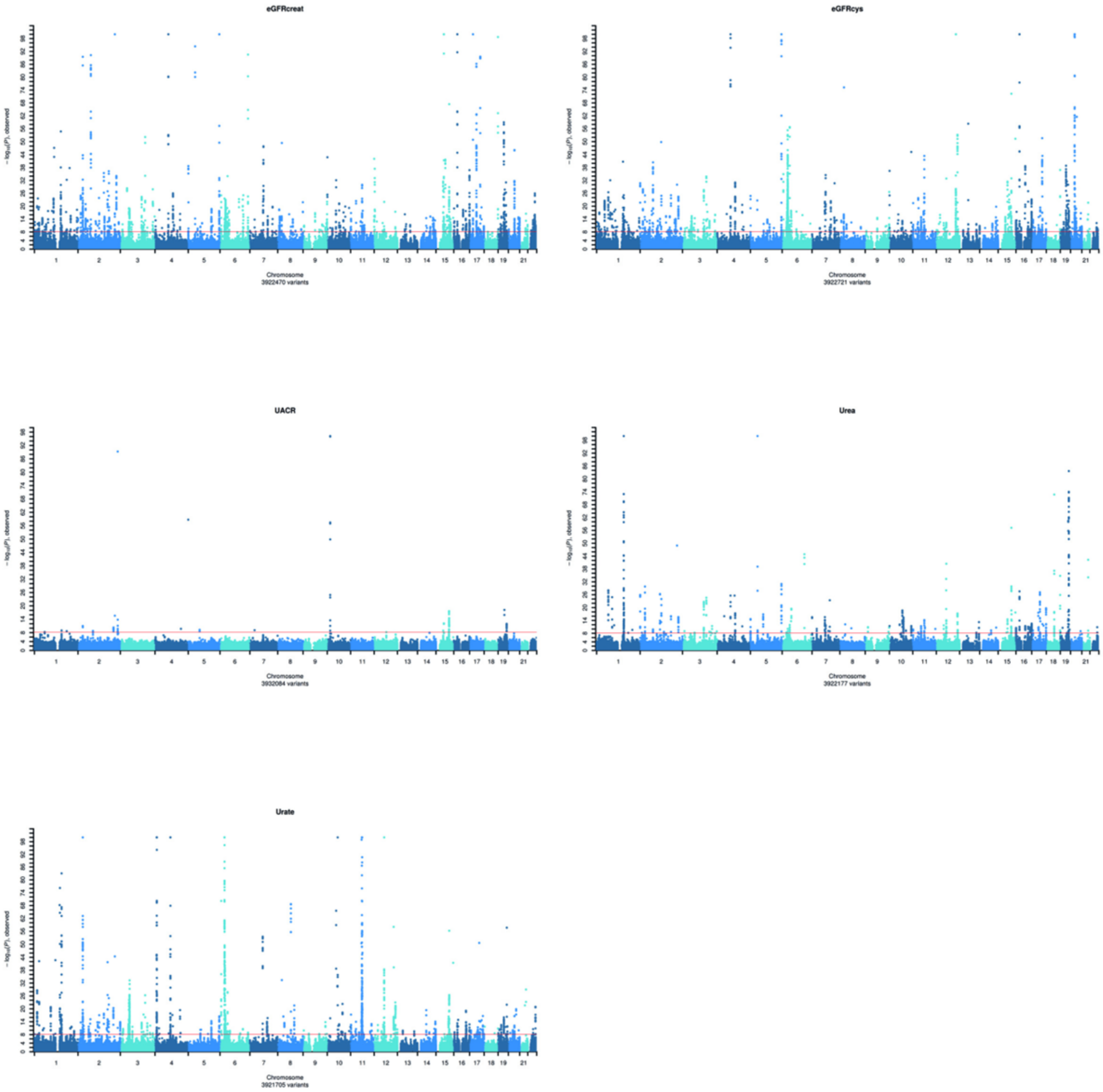

Manhattan plots of $-\log_{10}$ p-values of the single-variant association results by genomic position, filtered for a MAF>1.25e-5 (corresponding to a minor allele count ≥5). The figure caption shows the number of filtered and plotted variants. Two-sided p-values were obtained from linear mixed effect models (REGENIE) of effect allele dosage on phenotypes.

Supplementary Figure 3: Identified associations with rare variants are largely independent from associations with common variants

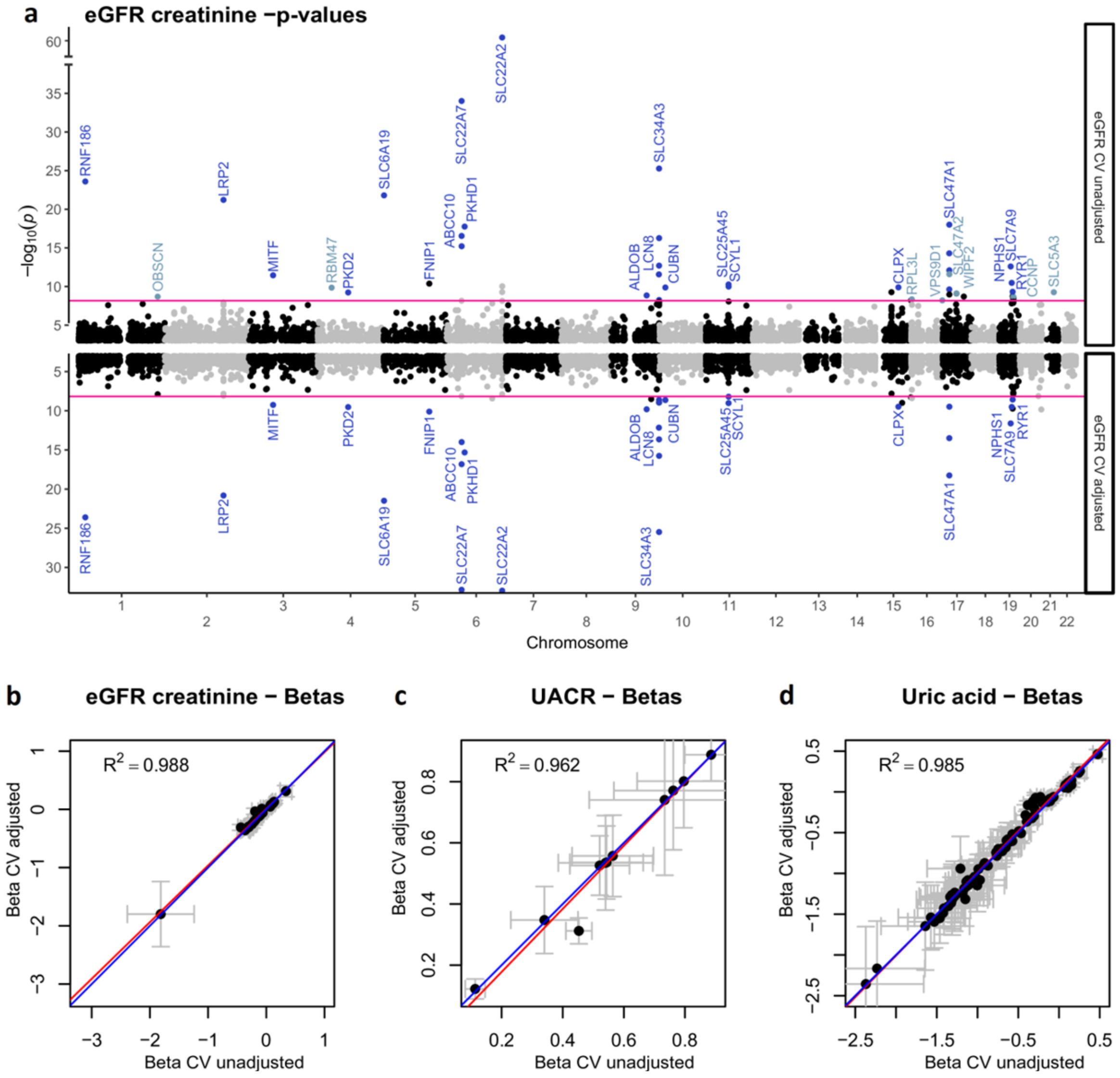

a, MIAMI plot showing genome-wide association of rare variants with eGFRcrea without adjusting for known association from common variants of previous GWAS studies (top) vs. the same analysis but additionally adjusting for them (bottom). Genes are labeled in dark-blue if they correspond to single-variants identified in the unadjusted and in the adjusted analysis and light-blue if they were only found in the unadjusted analysis. Two-sided p-values were obtained from linear mixed effect models (REGENIE) of effect allele dosage on phenotypes.

b-d, Comparison of effect sizes of common variant-unadjusted analysis with common variant-adjusted association analysis for eGFRcrea (**b**), UACR (**c**), and urate (**d**). All analyses were additionally adjusted for age, sex, and 40 principal components. Known common variants from GWAS accounted for 639 SNPs for eGFR, 63 SNPs for UACR, and 184 SNPs for

urate, error bars reflect 95% confidence intervals (±1.96·SE); see Methods for details. CV: common variant

Supplementary Figure 4. Distribution of UACR values by carrier genotype for variants in *CUBN*

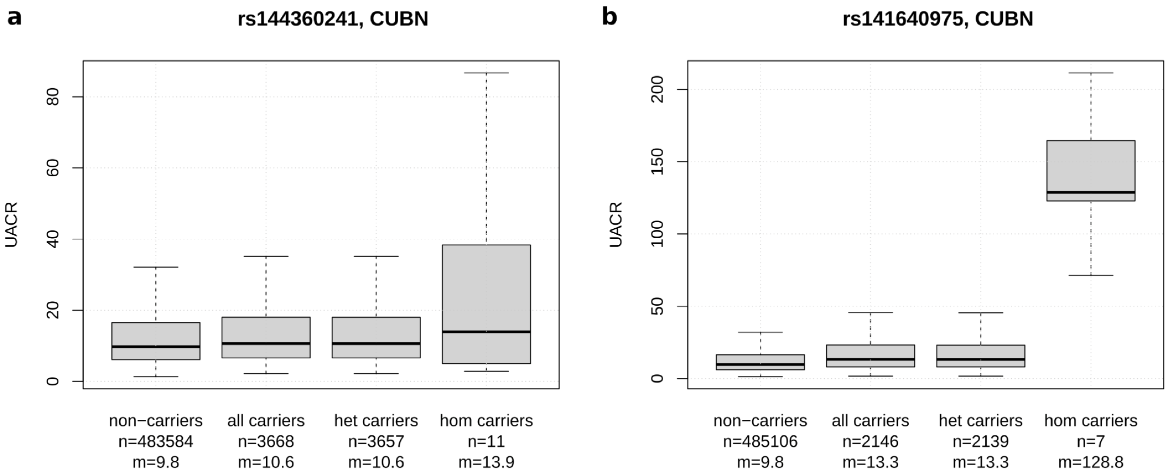

Boxplots showing raw (non INT transformed) UACR values for rs14436021 (**a**) and rs141640975 (**b**) in *CUBN* for non-carriers, all carriers, heterozygous carriers, and homozygous carriers of the alternative allele. Below each box, the number of samples (n) and the median value (m) is given. In both panels, the boxes represent the first to third quartile, the horizontal line the median, and the whiskers extend to 1.5 times the interquartile range.

Supplementary Figure 5: Quantile-quantile plots of gene-based test results

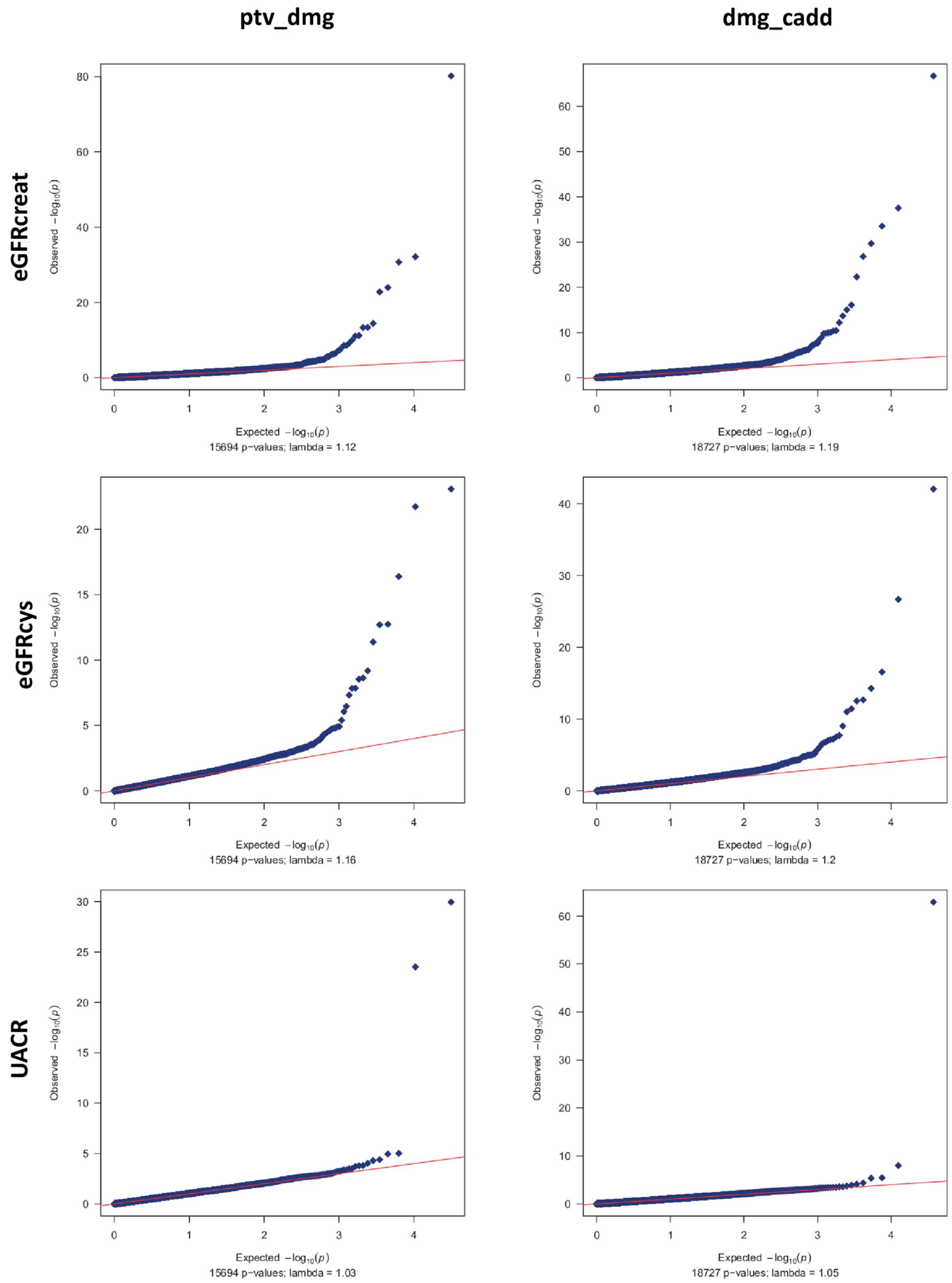

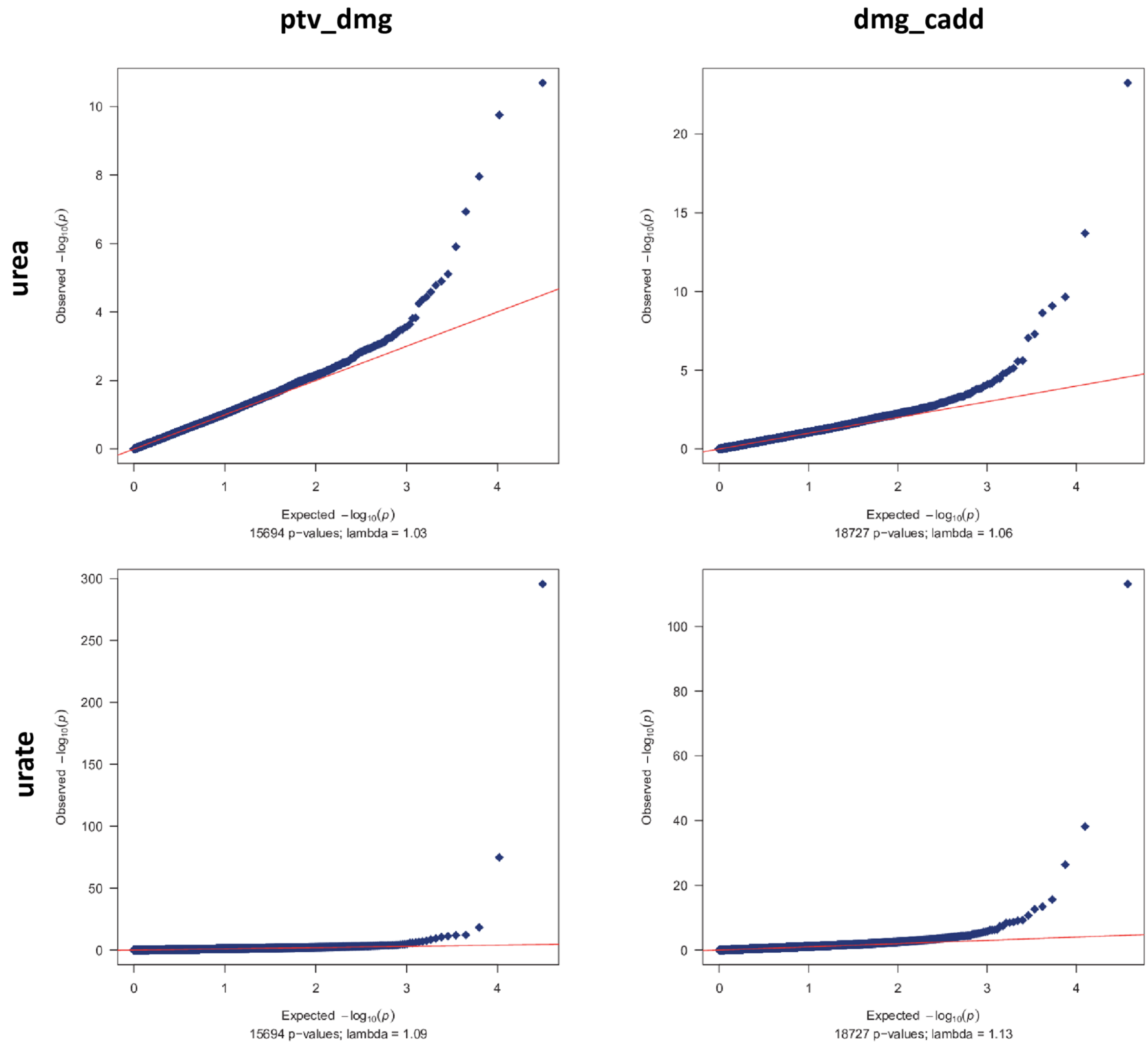

Plots of observed vs. expected –$\log_{10}$ association p-values for the five main phenotypes and the two masks “ptv_dmg” and “dmg_cadd”. The figure caption shows the number of tested genes and the inflation factor lambda. Two-sided p-values were obtained from linear mixed effect models (REGENIE) of effect allele dosage on phenotypes.

Supplementary Figure 6: Overlap plot between genes identified through gene-based testing with genes that when mutated can cause known monogenic kidney genes

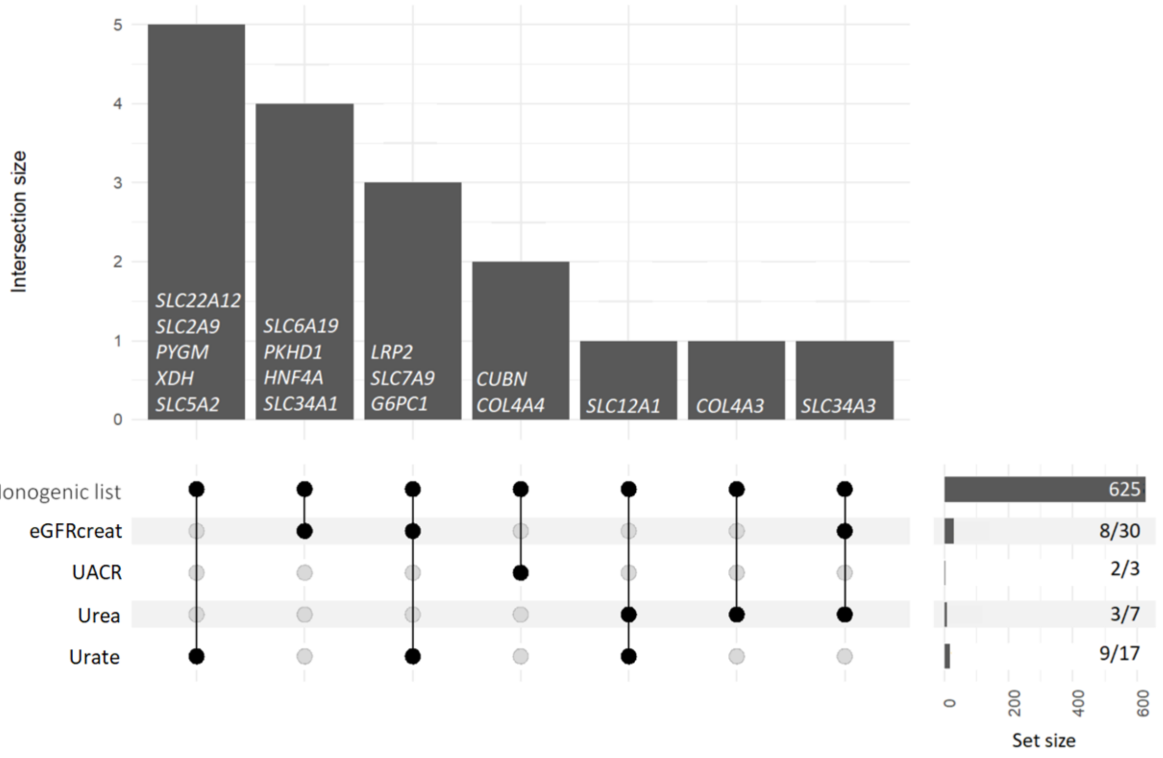

The bar plot in the right panel shows the total number of overlapping genes between four main phenotypes and the 625 known monogenic kidney genes. The left panel displays the overlapping phenotypes for each single gene. The upper panel lists the overlapping genes and gives its size.

Supplementary Figure 7: Add-one-in (blue) and leave-one-out (orange) plots for 56 significant gene – trait associations

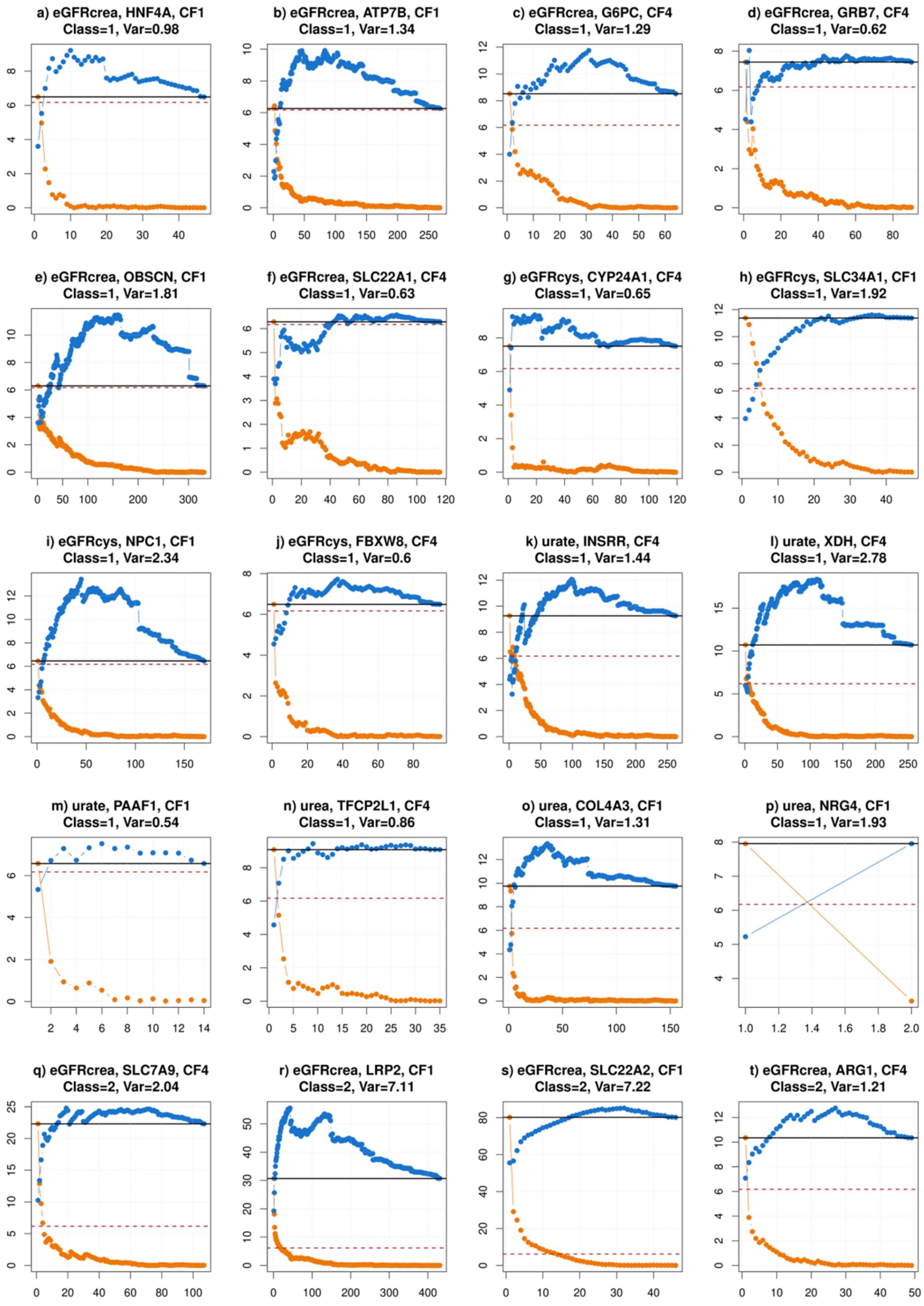

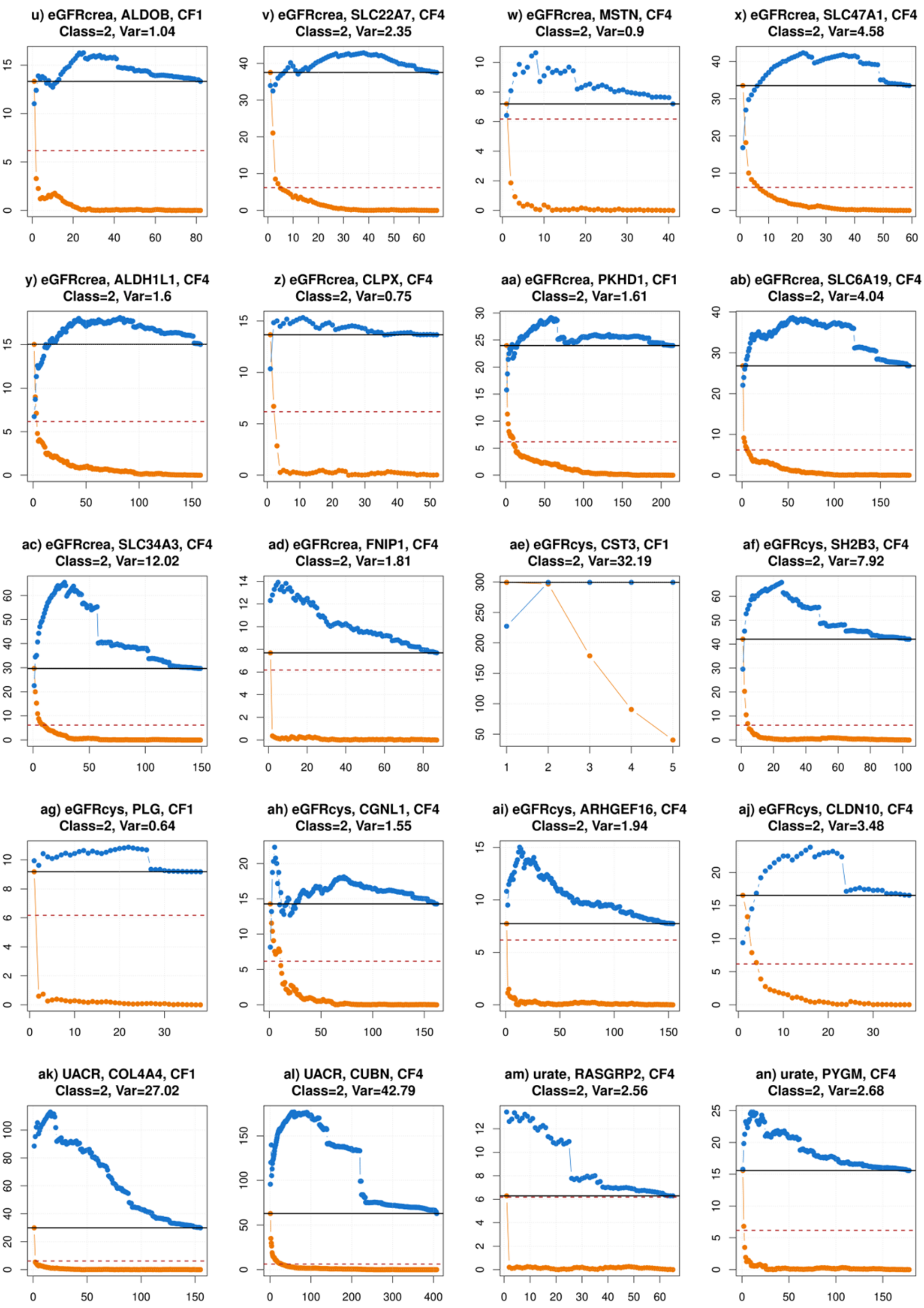
u) eGFRcrea, ALDOB, CF1
Class=2, Var=1.04
v) eGFRcrea, SLC22A7, CF4
Class=2, Var=2.35
w) eGFRcrea, MSTN, CF4
Class=2, Var=0.9
x) eGFRcrea, SLC47A1, CF4
Class=2, Var=4.58
y) eGFRcrea, ALDH1L1, CF4
Class=2, Var=1.6
z) eGFRcrea, CLPX, CF4
Class=2, Var=0.75
aa) eGFRcrea, PKHD1, CF1
Class=2, Var=1.61
ab) eGFRcrea, SLC6A19, CF4
Class=2, Var=4.04
ac) eGFRcrea, SLC34A3, CF4
Class=2, Var=12.02
ad) eGFRcrea, FNIP1, CF4
Class=2, Var=1.81
ae) eGFRcys, CST3, CF1
Class=2, Var=32.19
af) eGFRcys, SH2B3, CF4
Class=2, Var=7.92
ag) eGFRcys, PLG, CF1
Class=2, Var=0.64
ah) eGFRcys, CGNL1, CF4
Class=2, Var=1.55
ai) eGFRcys, ARHGEF16, CF4
Class=2, Var=1.94
aj) eGFRcys, CLDN10, CF4
Class=2, Var=3.48
ak) UACR, COL4A4, CF1
Class=2, Var=27.02
al) UACR, CUBN, CF4
Class=2, Var=42.79
am) urate, RASGRP2, CF4
Class=2, Var=2.56
an) urate, PYGM, CF4
Class=2, Var=2.68

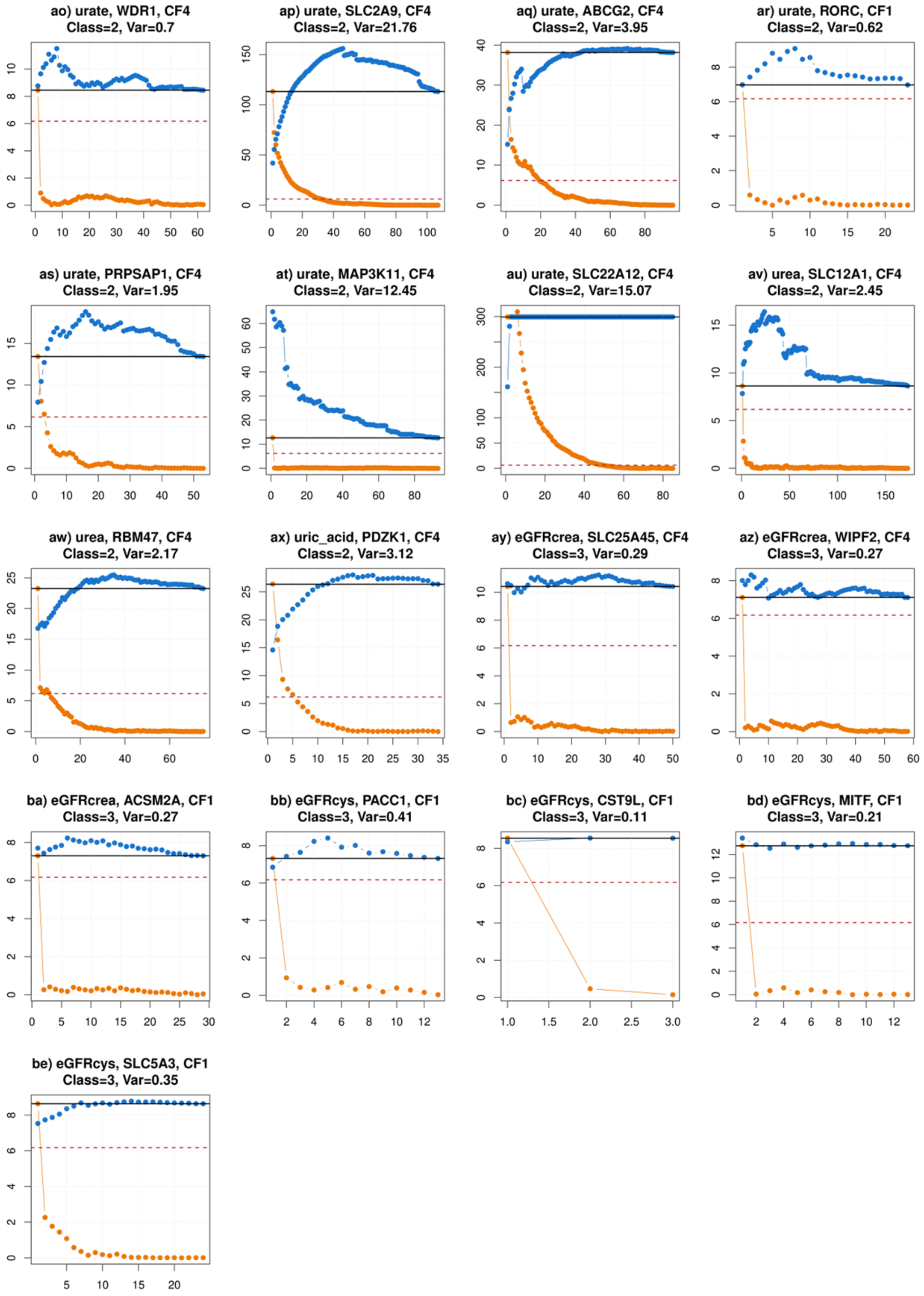
ao) urate, WDR1, CF4
Class=2, Var=0.7
ap) urate, SLC2A9, CF4
Class=2, Var=21.76
aq) urate, ABCG2, CF4
Class=2, Var=3.95
ar) urate, RORC, CF1
Class=2, Var=0.62
as) urate, PRPSAP1, CF4
Class=2, Var=1.95
at) urate, MAP3K11, CF4
Class=2, Var=12.45
au) urate, SLC22A12, CF4
Class=2, Var=15.07
av) urea, SLC12A1, CF4
Class=2, Var=2.45
aw) urea, RBM47, CF4
Class=2, Var=2.17
ax) uric_acid, PDZK1, CF4
Class=2, Var=3.12
ay) eGFRcrea, SLC25A45, CF4
Class=3, Var=0.29
az) eGFRcrea, WIPF2, CF4
Class=3, Var=0.27
ba) eGFRcrea, ACSM2A, CF1
Class=3, Var=0.27
bb) eGFRcys, PACC1, CF1
Class=3, Var=0.41
bc) eGFRcys, CST9L, CF1
Class=3, Var=0.11
bd) eGFRcys, MITF, CF1
Class=3, Var=0.21
be) eGFRcys, SLC5A3, CF1
Class=3, Var=0.35

In each subpanel, the –log10 gene burden p-value (y-axis) is plotted for each iteration *i* (x-axis) for the add-one-in (blue) and leave-one-out (orange) metric. The solid black line corresponds to the final gene p-value and the dotted red line to the significance threshold. Above each plot, the trait, gene and mask are given in the first line, and the assigned class and variance of the add-one-in metric in the second line. If a gene-trait association was significant with both masks, plots are only displayed for the mask that resulted in the lowest p-value. Two-sided p-values were obtained from linear regression models of mask variant risk allele dosage on phenotypes.

Supplementary Figure 8: Details of the gene-based analyses for *PKD1*

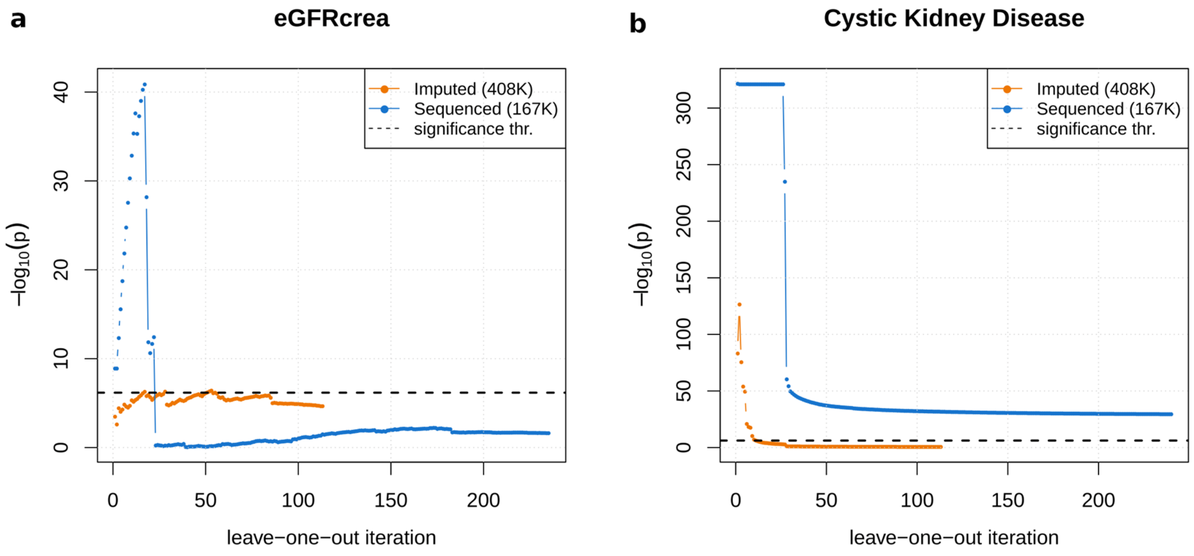

c

PKD1 Gene Analysis	MAF	eGFRcrea		Cystic Kidney Disease	
		p-value	n(var)	p-value	n(var)
**Imputed (408K)**	1%	2.0×10^{-5}	113	2.8×10^{-2}	120
	0.01%	8.9×10^{-3}	102	5.1×10^{-3}	49
**Sequenced (167K)**	1%	2.2×10^{-2}	235	1.4×10^{-30}	240
	0.01%	1.2×10^{-1}	222	6.2×10^{-121}	227

a-b, Leave-one-out plots for eGFRcrea (a) and cystic kidney disease (b) for the full dataset of imputed individuals (orange, 408K) and sequenced individuals only (blue 167K).

c, Gene burden p-values and number of variants for eGFRcrea and cystic kidney disease for two different MAF thresholds for the full dataset of imputed individuals and sequenced individuals only. Two-sided p-values were obtained from linear regression models of mask variant risk allele dosage on phenotypes.

Supplementary Figure 9: Pathway enrichment analyses

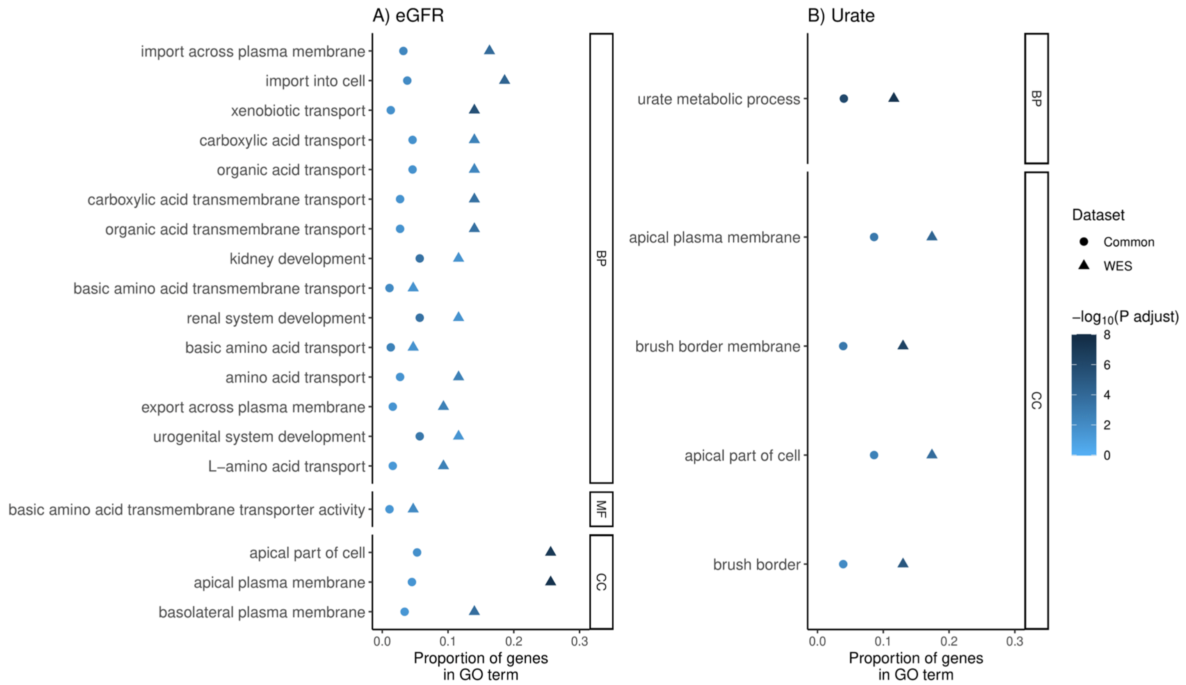

Proportion of enriched genes by GO (gene ontology) term, grouped by ontology: MF = molecular function, BP = biological process, CC = cellular component. Color-coding by $-\log_{10}$-transformed adjusted p-value (FDR 0.05). Symbols indicate the data set from which significantly associated genes for the enrichment analyses were identified (common GWAS[15–18] variants: circles, analyses WES (this study): triangles). Enrichment analyses for eGFR (**A**) and for serum urate (**B**). Two-sided p-values were obtained from linear mixed effect models (REGENIE) of effect allele dosage on phenotypes.

Supplementary Figure 10: Claudin-10b wildtype and frame-shift sequences.

```
NP_008915.1 (Claudin-10b)

  1 MASTASEIIAFMVSISGWVLVSSTLPTDYWKVSTIDGTVITTATYWANLWKACVTDSTGVSNCKDFPS
 69 MLALDGYIQACRGLMIAAVSLGFFGSIFALFGMKCTKVGGSDKAKAKIACLAGIVFILSGLCSMTGCS
136 LYANKITTEFFDPLFVEQKYELGAALFIGWAGASLCIIGGVIFCFSISDNNKTPRYTYNGATSVMSSR
205 TKYHGGEDFKTTNPSKQFDKNAYV                                          (wt)

205 TKYHGGEDFKTTNPSKQFEKCLCLKELAGKLPLEFVIKANCSQNDPIKALP   (fs; present manuscript)

205 TKYHGGEDFKTTNLQNSLIKMLMSKRARGQAAS               (fs(SA); Alzahrani et al., 2021)
```

Compared to the claudin-10b wildtype (wt) sequence, deletion of two bases (c.668_669delAT) in CLDN10b and the resulting frame-shift (fs) causes the replacement of the six C-terminal amino acids of the claudin-10b wt sequence (DKNAYV) by 33 amino acids (EKCLCLKELAGKLPLEFVIKANCSQNDPIKALP) in claudin-10b fs.

In a recently published frame-shift mutation found in a cohort of Saudi Arabian HELIX syndrome patients (fs(SA); Alzahrani et al., 2021), only one base was deleted (c.653delC in CLDN10b), replacing the 11 C-terminal amino acids of the claudin-10b wt sequence (PSKQFDKNAYV) by 20 amino acids (LQNSLIKMLMSKRARGQAAS). Please note that with respect to the reference sequence, the nucleotide sequence in these patients additionally contained a SNP (rs1325774) in the region that would be 3' UTR in the wt sequence (c.698T>G).

Both fs proteins lack the C-terminal pdz binding motif of the wt claudin-10b (underlined).

Supplementary Figure 11: Trans-interaction of claudin-10b wt and fs in HEK 293 cell-cell contacts

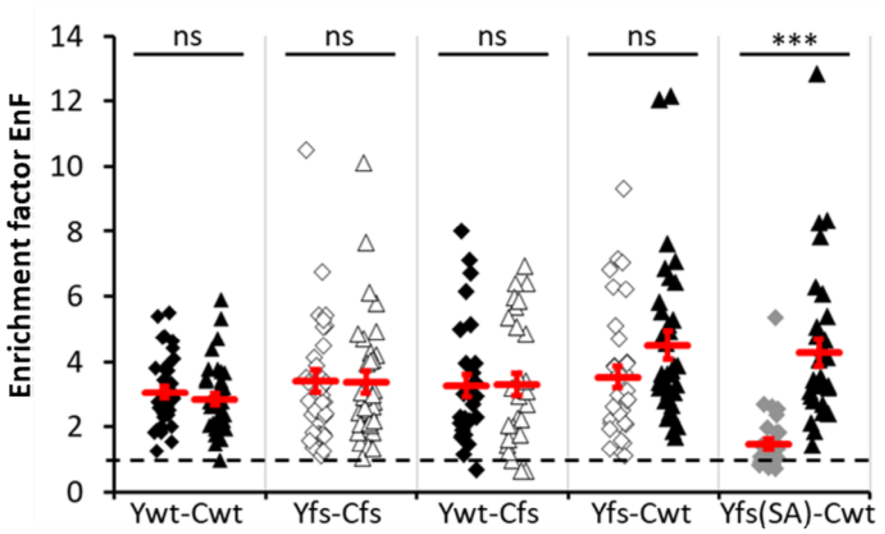

To evaluate the ability for *trans*-interaction, HEK 293 cells were co-transfected with different combinations of YFP(Y; diamonds)- or CFP(C; triangles)-tagged wildtype (wt; black symbols) or frame-shift claudin-10b (fs, present study, white symbols; fs(SA), mutant described by Alzahrani et al., 2021, grey symbols). Contact enrichment as indicator for *trans*-interaction was evaluated as described in detail in **Supplementary Figure 14**. It was similar for all constructs and independent of the combination (wt-wt, fs-fs, wt-fs), and of their tag (YFP and CFP-tagged constructs were evaluated before and after bleaching, respectively). In contrast, fs(SA) only showed marginal contact enrichment (Enf-values ≈1, dashed line). YFP-wt – CFP-wt, n=37 from m = 5 independent transfections; YFP-fs – CFP-fs, n=36, m=5; YFP-wt – CFP-fs, n=29, m=4; YFP-fs – CFP-wt, n=34, m=4; YFP-fs(SA) – CFP-wt, n=32, m=4. Red lines indicate mean Enrichment factor ± SEM; unpaired, two-sided Student's t-test; ns, not significant; ***, p=$3.3 \cdot 10^{-8}$.

Supplementary Figure 12: Imputation quality in 10,000 validation samples and 2,191,400 variants for different filtering thresholds

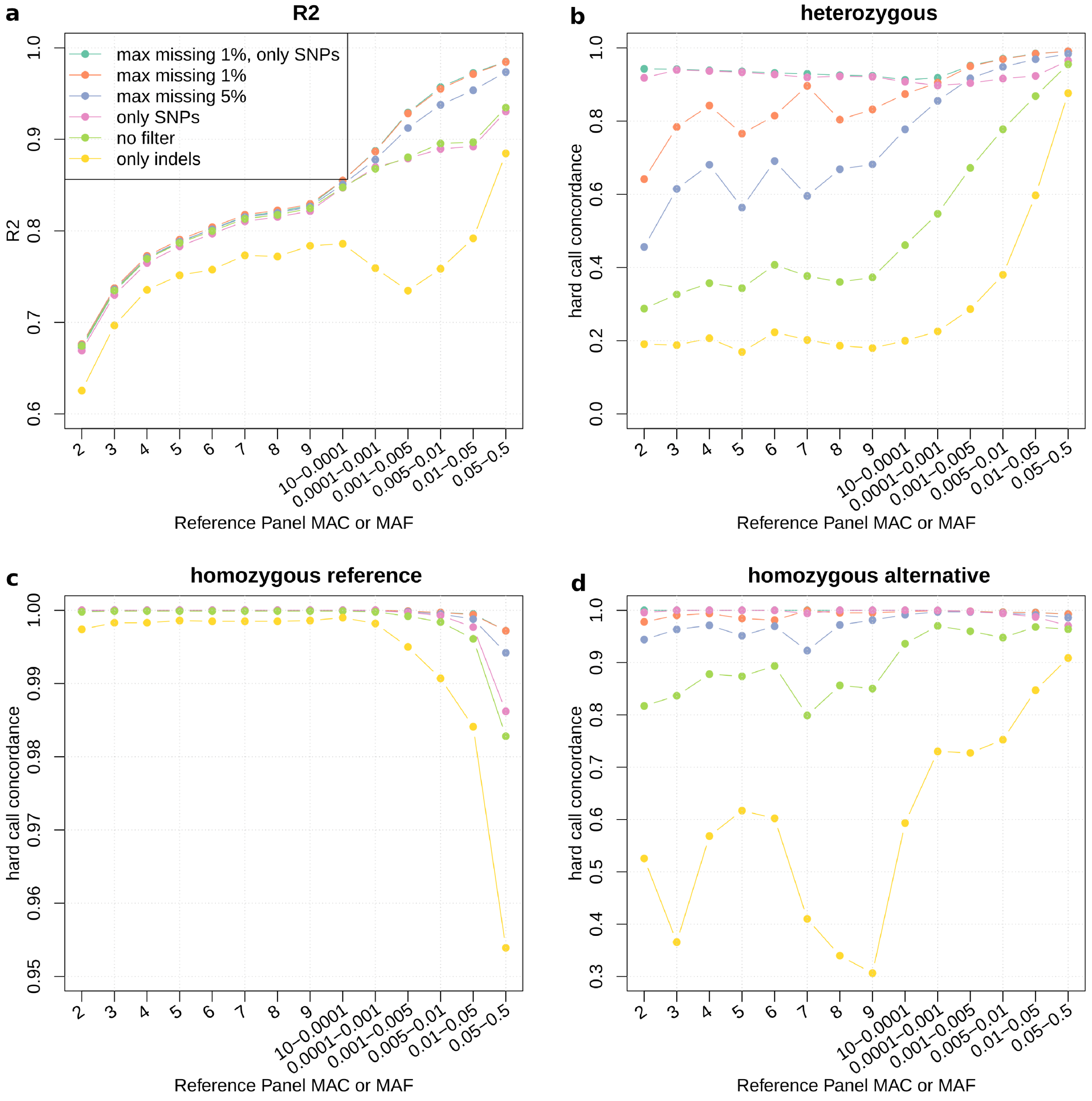

a, Mean squared correlation (R2) of sequenced genotypes with imputed dosages for different variant missingness thresholds and different groups of variants.

b-d, Mean genotype concordance of the hard call imputed genotypes with sequenced data for heterozygous calls (**b**), homozygous reference calls (**c**), and homozygous alternative calls (**d**) for different variant missingness thresholds and different groups of variants.

Supplementary Figure 13: Phenotype correlation

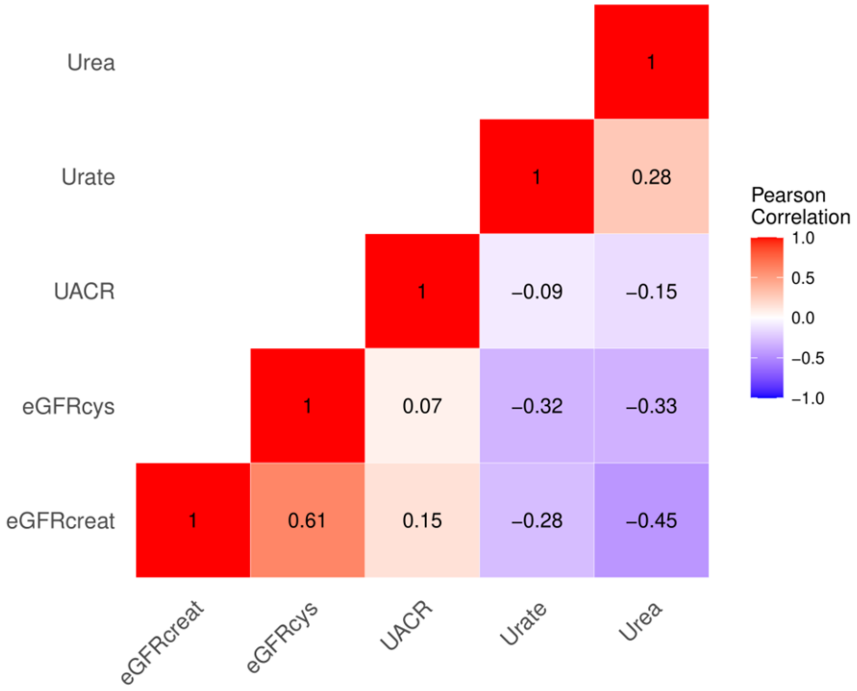

Heatmap showing pairwise Pearson's correlation coefficients for main phenotypes.

Supplementary Figure 14: FRET assay to quantify cis-interaction of tight junction proteins

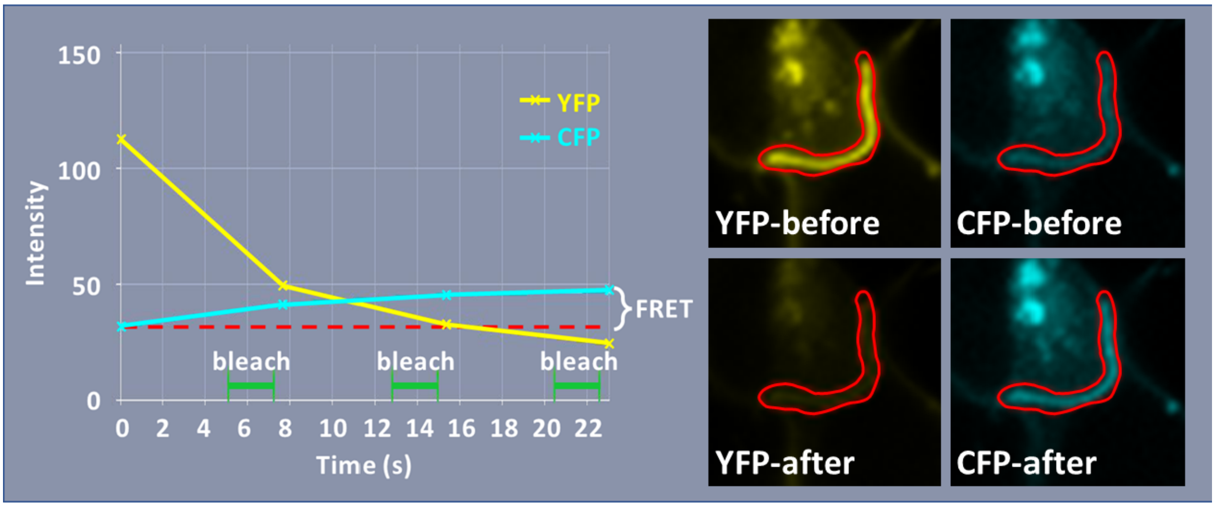

YFP and CFP fluorescence signals of two adjacent cells co-transfected with YFP- and CFP-tagged wt CLDN10b are shown before and after acceptor bleach. Using the Zeiss ZEN software, a region of interest (ROI) was drawn to encircle the cell – cell contact zone. The intensity plot shows the average YFP and CFP intensities within the ROI at four different time points. Time point one (t_1) is before acceptor bleach, time points two to four ($t_2 – t_4$) after three consecutive bleachings (as indicated by the green lines). As YFP intensity decreases, CFP intensity increases. FRET efficiency was evaluated as the relative increase in CFP fluorescence intensity: FRET efficiency = $(I_a − I_b)/I_a \times 100\%$. I_b, CFP fluorescence intensity at t_1; I_a, CFP fluorescence intensity at t_4.

Supplementary Figure 15: Enrichment assay to quantify trans-interaction of tight junction proteins.

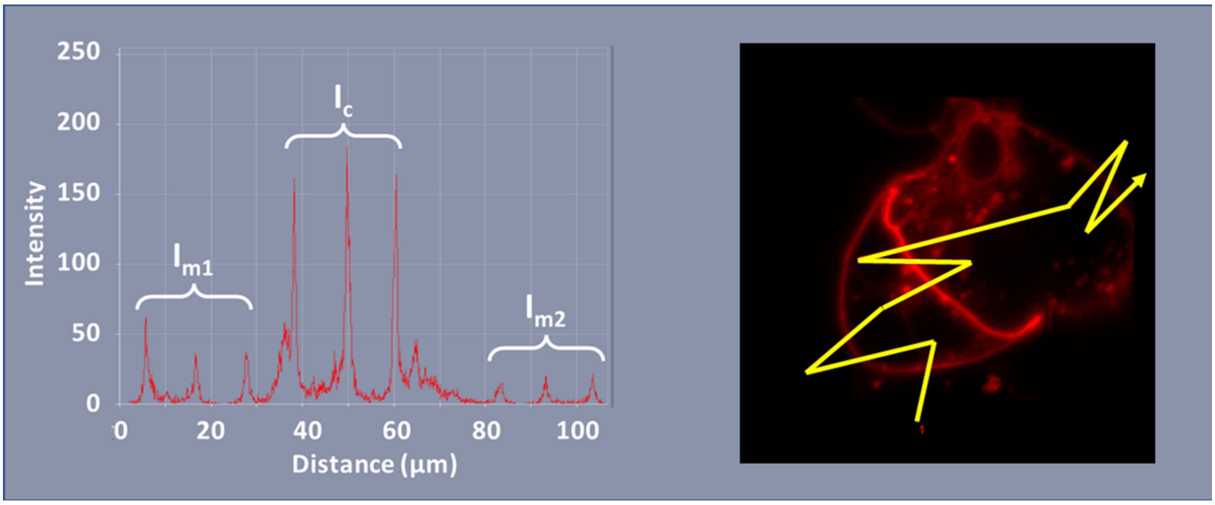

Fluorescence signals of two adjacent cells transfected with YFP-tagged wt CLDN10b are shown. Using the Zeiss ZEN software, a line was drawn to cross the cell membranes outside the cell – cell contact as well as within the contact zone three times each. The intensity plot consequently shows three peaks for the intensities of the membrane signals of cell one and cell two, and for the contact, respectively. The average of the respective peak amplitudes (I_{m1}, I_{m2}, I_c) were calculated and the enrichment factor EnF was calculated as EnF = $I_c/(I_{m1} + I_{m2})$.

Supplementary References

1. Jefferson, J. A. *et al.* Autosomal dominant Alport syndrome linked to the type IV collage alpha 3 and alpha 4 genes (COL4A3 and COL4A4). *Nephrol. Dial. Transplant. Off. Publ. Eur. Dial. Transpl. Assoc. - Eur. Ren. Assoc.* **12**, 1595–1599 (1997).
2. Barua, M. & Paterson, A. D. Population-based studies reveal an additive role of type IV collagen variants in hematuria and albuminuria. *Pediatr. Nephrol. Berl. Ger.* **37**, 253–262 (2022).
3. Yao, T. *et al.* Integration of Genetic Testing and Pathology for the Diagnosis of Adults with FSGS. *Clin. J. Am. Soc. Nephrol. CJASN* **14**, 213–223 (2019).
4. Malone, A. F. *et al.* Rare hereditary COL4A3/COL4A4 variants may be mistaken for familial focal segmental glomerulosclerosis. *Kidney Int.* **86**, 1253–1259 (2014).
5. Storey, H., Savige, J., Sivakumar, V., Abbs, S. & Flinter, F. A. COL4A3/COL4A4 mutations and features in individuals with autosomal recessive Alport syndrome. *J. Am. Soc. Nephrol. JASN* **24**, 1945–1954 (2013).
6. Sinnott-Armstrong, N. *et al.* Genetics of 35 blood and urine biomarkers in the UK Biobank. *Nat. Genet.* **53**, 185–194 (2021).
7. Barton, A. R., Sherman, M. A., Mukamel, R. E. & Loh, P.-R. Whole-exome imputation within UK Biobank powers rare coding variant association and fine-mapping analyses. *Nat. Genet.* **53**, 1260–1269 (2021).
8. Backman, J. D. *et al.* Exome sequencing and analysis of 454,787 UK Biobank participants. *Nature* **599**, 628–634 (2021).
9. Li, S. *et al.* The GLUT9 gene is associated with serum uric acid levels in Sardinia and Chianti cohorts. *PLoS Genet.* **3**, e194 (2007).
10. Kolz, M. *et al.* Meta-analysis of 28,141 individuals identifies common variants within five new loci that influence uric acid concentrations. *PLoS Genet.* **5**, e1000504 (2009).
11. Tin, A. *et al.* Genome-wide association study for serum urate concentrations and gout among African Americans identifies genomic risk loci and a novel URAT1 loss-of-function allele. *Hum. Mol. Genet.* **20**, 4056–4068 (2011).
12. Tin, A. *et al.* Large-scale whole-exome sequencing association studies identify rare functional variants influencing serum urate levels. *Nat. Commun.* **9**, 4228 (2018).
13. Pizzamiglio, C., Mahroo, O. A., Khan, K. N., Patasin, M. & Quinlivan, R. Phenotype and genotype of 197 British patients with McArdle disease: An observational single-centre study. *J. Inherit. Metab. Dis.* **44**, 1409–1418 (2021).
14. Sulem, P. *et al.* Identification of low-frequency variants associated with gout and serum uric acid levels. *Nat. Genet.* **43**, 1127–1130 (2011).
15. Wuttke, M. *et al.* A catalog of genetic loci associated with kidney function from analyses of a million individuals. *Nat. Genet.* **51**, 957–972 (2019).
16. Stanzick, K. J. *et al.* Discovery and prioritization of variants and genes for kidney function in >1.2 million individuals. *Nat. Commun.* **12**, 4350 (2021).
17. Tin, A. *et al.* Target genes, variants, tissues and transcriptional pathways influencing human serum urate levels. *Nat. Genet.* **51**, 1459–1474 (2019).
18. Teumer, A. *et al.* Genome-wide association meta-analyses and fine-mapping elucidate pathways influencing albuminuria. *Nat. Commun.* **10**, 4130 (2019).
