# Supplementary material for: Imputation-powered whole-exome analysis identifies genes associated with kidney function and disease in the UK Biobank
Source: Nat Commun. 2023 Mar 9;14:1287. doi: 10.1038/s41467-023-36864-8 (PMC9995463; doi:10.1038/s41467-023-36864-8)
Supplement: Supplementary file 2 — Description of Additional Supplementary Files [file 41467_2023_36864_MOESM2_ESM.pdf]

Description of Additional Supplementary Files

File Name: Supplementary Data 1

Description: Filtered Exome-wide association results for the five main phenotypes eGFR-creatinine, eGFR-cystatin C, UACR, urate and urea. Two-sided p-values were obtained from linear mixed effect models (REGENIE) of effect allele dosage on phenotypes.

Includes results with minor allele frequency <1%, minor allele count of >=5, imputation INFO score > 0.5, association p-value <6.78e-9, impact >= LOW and non-synonymous (Methods). Variant in LD are annotated with their LD partners and r^2. For example, two variants on chromosome 19 associated with the UACR, p.Ser937Asn (rs201194276) in *NPHS1* and p.Val175Met (rs376992247) in *IGFLR1* were highly correlated (r^2=0.87), likely reflecting the same signal. Rare damaging variants in *NPHS1* are a known cause of congenital Nephrotic Syndrome of the Finnish type, a disease featuring highly elevated UACR levels, implicating p.Ser937Asn as the likely causal allele.

Chr = chromosome, Pos_b38 = genomic position (build 38), Ref_Allele = reference allele, Effect_Allele = effect allele, Freq_effect_all = frequency of the effect allele, MAC = minor allele count, Info = imputation info score, N = number of samples, Beta = coefficient of association estimate, SE = standard error of Beta, Pval = p-value, gnomAD_AF = maximum allele frequency in the GnomAD exome database, gnomAD_AF_NFE = allele frequency in non-Finnish Europeans in the GnomAD exome database, REVEL = REVEL score, CADD_PHRED = CADD score, phred-scaled, LRT = likelihood-ratio test for deleteriousness, M.CAP = Mendelian Clinically Applicable Pathogenicity Score, MetaSVM = meta-score incorporating multiple in-silico scores, FathMM.XF = Prediction score of pathogenic point mutations, ClinVar_Clnsig = clinical significance from the ClinVar database, clinvar_Trait = associated trait from the ClinVar database, Loftee_LoF = loss-of-function (LoF) rating of the LoFtee tool; HC = high confidence LoF, LC = low confidence; CKDGen_Known = variant identifier (rsid) of known common GWAS locus in proximity (+/-500 kb), LD = other significantly associated variants in LD, together with r^2 value. beta/SE/pval columns in the end = lookup of association statistics for other association (CKD, other phenotypes). UA = urinary albumin, UC = urinary creatinine, MA = microalbuminuria

File Name: Supplementary Data 2

Description: Table 1 with additional columns. Genes associated with more than one kidney function measure (eGFRcreat, eGFRcys, urea) and direction-consistent association with CKD from ExWAS and gene-based tests are listed. Two-sided p-values were obtained from linear regression models of mask variant risk allele dosage on phenotypes.

*1 “x” indicates presence in single variant ExWAS or association test that group alleles in a gene (gene-level burden test)

*2 Kidney cell type in which the gene is specifically expressed. PT = proximal tubule, LOH = loop of Henle, DVR = descending vasa recta endothelium, EP = epithelial progenitor, PC = principal cell, PE = pelvic epithelium, POD = podocyte, GE = glomerular endothelium, CT = connecting tubule

*3 dbSNP ids of variants of known eGFR GWAS loci based on Stancick et al. within ± 100kb of the variant position or gene start/end.

*4 aut-rec = autosomal recessive, aut-dom: autosomal dominant

File Name: Supplementary Data 3
Description: Gene-based testing results. One table sheet per mask (ptv_dmg and dmg_cadd). Filtered for $p<6.72\times10^{-7}$. Two-sided p-values were obtained from linear regression models of mask variant risk allele dosage on phenotypes. Grouped by phenotype. Each gene may appear multiple times if associated with multiple phenotypes. Lists p-value, effect size (beta) and standard error (SE) from the Burden test. The cumulative minor allele frequency (cMAF) represents all variants grouped by the mask in the given gene. nSNPs = number of variants examined in the Burden test. SKAT_p = p-value of SKAT test.

File Name: Supplementary Data 4
Description: Single variants included in group-based testing. Lists contributing single variants for each of the tested phenotypes and for each tested mask. Includes variants for all significant genes. Not filtered for single-variant association p-value to list all discovered variants in the respective genes. Two-sided p-values were obtained from linear mixed effect models (REGENIE) of effect allele dosage on phenotypes. Columns include variant annotation information (shortcuts: Supplementary Data 1).

File Name: Supplementary Data 5
Description: Phenome-wide association study of associated kidney function genes. Phenome-wide association study of significant single variant and gene-based testing associations of kidney function genes with other phenotypes in the UK Biobank. Filtered for association P-value < 5×10^{-8}. P-values are shown as reported by the respective studies. Requires more than 5 cases with qualifying variants (for binary traits) or more than 5 individuals with qualifying variants (for quantitative traits).

File Name: Supplementary Data 6
Description: Pathway enrichment analyses. GO terms pathway enrichment analyses per phenotype (eGFR, urate) for common GWAS genes (left part) and WES genes (right part). GO = gene ontology. BP = biological process, CC = cellular component, MF = molecular function. Gene ratio: number of associated genes contained in gene set vs. total number of genes. Adj. p-value = adjusted p-value (FDR 0.05). Two-sided p-values were obtained from linear regression models of mask variant risk allele dosage on phenotypes.

File Name: Supplementary Data 7
Description: Number of variants or genotypes used for the evaluation of imputation accuracy. Data used in Supplementary Figure 12.
