# Supplementary material for: Imputation-powered whole-exome analysis identifies genes associated with kidney function and disease in the UK Biobank
Source: Nat Commun. 2023 Mar 9;14:1287. doi: 10.1038/s41467-023-36864-8 (PMC9995463; doi:10.1038/s41467-023-36864-8)
Supplement: Supplementary file 4 — Reporting Summary [file 41467_2023_36864_MOESM4_ESM.pdf]

nature portfolio

Corresponding author(s): Matthias Wuttke, Christian Fuchsberger

Last updated by author(s): Feb 2, 2023

Reporting Summary

Nature Portfolio wishes to improve the reproducibility of the work that we publish. This form provides structure for consistency and transparency in reporting. For further information on Nature Portfolio policies, see our Editorial Policies and the Editorial Policy Checklist.

Statistics

For all statistical analyses, confirm that the following items are present in the figure legend, table legend, main text, or Methods section.

n/a	Confirmed	
☐	☒	The exact sample size (*n*) for each experimental group/condition, given as a discrete number and unit of measurement
☐	☒	A statement on whether measurements were taken from distinct samples or whether the same sample was measured repeatedly
☐	☒	The statistical test(s) used AND whether they are one- or two-sided<br>*Only common tests should be described solely by name; describe more complex techniques in the Methods section.*
☐	☒	A description of all covariates tested
☐	☒	A description of any assumptions or corrections, such as tests of normality and adjustment for multiple comparisons
☐	☒	A full description of the statistical parameters including central tendency (e.g. means) or other basic estimates (e.g. regression coefficient) AND variation (e.g. standard deviation) or associated estimates of uncertainty (e.g. confidence intervals)
☐	☒	For null hypothesis testing, the test statistic (e.g. *F*, *t*, *r*) with confidence intervals, effect sizes, degrees of freedom and *P* value noted<br>*Give P values as exact values whenever suitable.*
☒	☐	For Bayesian analysis, information on the choice of priors and Markov chain Monte Carlo settings
☐	☒	For hierarchical and complex designs, identification of the appropriate level for tests and full reporting of outcomes
☐	☒	Estimates of effect sizes (e.g. Cohen's *d*, Pearson's *r*), indicating how they were calculated

Our web collection on statistics for biologists contains articles on many of the points above.

Software and code

Policy information about availability of computer code

Data collection	No tool was used to collect data.
Data analysis	Details are described in the methods. Code for the central tools is available online (REGENIE v2.0.2: https://rgcgithub.github.io/regenie, PLINK: https://www.cog-genomics.org/plink/). Custom scripts and codes available at https://github.com/genepi-freiburg/seqmeta

For manuscripts utilizing custom algorithms or software that are central to the research but not yet described in published literature, software must be made available to editors and reviewers. We strongly encourage code deposition in a community repository (e.g. GitHub). See the Nature Portfolio guidelines for submitting code & software for further information.

Data

Policy information about availability of data

All manuscripts must include a data availability statement. This statement should provide the following information, where applicable:

- Accession codes, unique identifiers, or web links for publicly available datasets
- A description of any restrictions on data availability
- For clinical datasets or third party data, please ensure that the statement adheres to our policy

We used publicly available individual-level genotype and phenotype data from the UK Biobank (https://biobank.ndph.ox.ac.uk/showcase/). Our results are shared with the community using a comprehensive online resource: https://ckdgen-ukbb.gm.eurac.edu/

Human research participants

Policy information about studies involving human research participants and Sex and Gender in Research.

Reporting on sex and gender	In our analyses, self-reported biological sex was used.
Population characteristics	Analogous to other studies, 408,511 participants that were part of the white British ancestry subset according to the genotype quality control of the UK Biobank were included in this study. Study characteristics are described in detail by Bycroft et al, Nature 2018 (https://www.nature.com/articles/s41586-018-0579-z).
Recruitment	Please see Bycroft et al., Nature 2018
Ethics oversight	This work was conducted within approved UK Biobank application numbers 20272 and 64806.

Note that full information on the approval of the study protocol must also be provided in the manuscript.

Field-specific reporting

Please select the one below that is the best fit for your research. If you are not sure, read the appropriate sections before making your selection.

☒ Life sciences ☐ Behavioural & social sciences ☐ Ecological, evolutionary & environmental sciences

For a reference copy of the document with all sections, see nature.com/documents/nr-reporting-summary-flat.pdf

Life sciences study design

All studies must disclose on these points even when the disclosure is negative.

Sample size	Sample size for Claudin-10 FRET and Enrichment studies was chosen according to our previous publications that involved the same wt controls but different mutations (Klar J, Piontek J, Milatz S, Tariq M, Jameel M, Breiderhoff T, Schuster J, Fatima A, Asif M, Sher M, Mäbert K, Fromm A, Baig SM, Günzel D, Dahl N (2017) Altered paracellular cation permeability due to a rare CLDN10B variant causes anhidrosis and kidney damage. Plos Genet. 13(7): e1006897, 10.1371/journal.pgen.1006897; Sewerin S, Piontek J, Schönauer R, Grunewald S, Rauch A, Neuber S, Bergmann C, Günzel D*, Halbritter J* (*shared last authorship) (2022) Defective claudin-10 causes a novel variation of HELIX syndrome through compromised tight junction strand assembly. Genes & Dis. 9(5): 1301-1314, doi: 10.1016/j.gendis.2021.06.006)
Data exclusions	No data were excluded.
Replication	Data originate from 4 to five independent transfections per condition.
Randomization	Cells were dissociated into single cell suspension. Cells from each suspension were seeded on a set of cover slips which were then randomly transfected with the different combinations of plasmids containing wt and mutated claudin constructs.
Blinding	Blinding was not possible, because cell-cell contacts differed visibly, depending in the combinations of constructs used.

Reporting for specific materials, systems and methods

We require information from authors about some types of materials, experimental systems and methods used in many studies. Here, indicate whether each material, system or method listed is relevant to your study. If you are not sure if a list item applies to your research, read the appropriate section before selecting a response.

Materials & experimental systems

n/a	Involved in the study	
☒	☐	Antibodies
☐	☒	Eukaryotic cell lines
☒	☐	Palaeontology and archaeology
☒	☐	Animals and other organisms
☒	☐	Clinical data
☒	☐	Dual use research of concern

Methods

n/a	Involved in the study	
☒	☐	ChIP-seq
☒	☐	Flow cytometry
☒	☐	MRI-based neuroimaging

Eukaryotic cell lines

Policy information about cell lines and Sex and Gender in Research

Cell line source(s)	HEK 293: ATCC
Authentication	Cell line authentication was last carried out in 2018 by eurofins: DNA was isolated separately from the samples. Genetic characteristics were determined by PCR-single-locus-technology. 21 independent PCR-systems Amelogenin, D3S1358, D1S1656, D6S1043, D13S317, Penta E, D16S539, D18S51, D2S1338, CSF1PO, Penta D, TH01, vWA, D21S11, D7S820, D5S818, TPOX, D8S1179, D12S391, D19S433 and FGA were investigated (Promega, PowerPlex 21 PCR Kit). In parallel, positive and negative controls were carried out yielding correct results.
Mycoplasma contamination	All cell cultures in the lab are tested by PCR at regular intervals and only negatively tested cells are used.
Commonly misidentified lines (See ICLAC register)	No commonly misidentified cell lines were used in the study.
